# Mechanism of lactic acidemia-promoted pulmonary endothelial cells death in sepsis: role for CIRP-ZBP1-PANoptosis pathway

**DOI:** 10.1186/s40779-024-00574-z

**Published:** 2024-10-28

**Authors:** Ting Gong, Qing-De Wang, Patricia A. Loughran, Yue-Hua Li, Melanie J. Scott, Timothy R. Billiar, You-Tan Liu, Jie Fan

**Affiliations:** 1 Department of Surgery, University of Pittsburgh School of Medicine, Pittsburgh, PA 15213 USA; 2 Department of Anesthesiology, Shenzhen Hospital of Southern Medical University, Shenzhen, 518110 Guangdong China; 3 McGowan Institute for Regenerative Medicine, University of Pittsburgh, Pittsburgh, PA 15219 USA; 4 Department of Immunology, University of Pittsburgh School of Medicine, Pittsburgh, PA 15213 USA; 5 Research and Development, Veterans Affairs Pittsburgh Healthcare System, Pittsburgh, PA 15240 USA

**Keywords:** Extracellular cold-inducible RNA-binding protein (eCIRP), PANoptosis, ZBP1, Ubiquitination, Sepsis-induced acute lung injury (ALI)

## Abstract

**Background:**

Sepsis is often accompanied by lactic acidemia and acute lung injury (ALI). Clinical studies have established that high serum lactate levels are associated with increased mortality rates in septic patients. We further observed a significant correlation between the levels of cold-inducible RNA-binding protein (CIRP) in plasma and bronchoalveolar lavage fluid (BALF), as well as lactate levels, and the severity of post-sepsis ALI. The underlying mechanism, however, remains elusive.

**Methods:**

C57BL/6 wild type (WT), *Casp8*^*−/−*^, *Ripk3*^*−/−*^, and *Zbp1*^*−/−*^ mice were subjected to the cecal ligation and puncture (CLP) sepsis model. In this model, we measured intra-macrophage CIRP lactylation and the subsequent release of CIRP. We also tracked the internalization of extracellular CIRP (eCIRP) in pulmonary vascular endothelial cells (PVECs) and its interaction with Z-DNA binding protein 1 (ZBP1). Furthermore, we monitored changes in ZBP1 levels in PVECs and the consequent activation of cell death pathways.

**Results:**

In the current study, we demonstrate that lactate, accumulating during sepsis, promotes the lactylation of CIRP in macrophages, leading to the release of CIRP. Once eCIRP is internalized by PVEC through a Toll-like receptor 4 (TLR4)-mediated endocytosis pathway, it competitively binds to ZBP1 and effectively blocks the interaction between ZBP1 and tripartite motif containing 32 (TRIM32), an E3 ubiquitin ligase targeting ZBP1 for proteasomal degradation. This interference mechanism stabilizes ZBP1, thereby enhancing ZBP1-receptor-interacting protein kinase 3 (RIPK3)-dependent PVEC PANoptosis, a form of cell death involving the simultaneous activation of multiple cell death pathways, thereby exacerbating ALI.

**Conclusions:**

These findings unveil a novel pathway by which lactic acidemia promotes macrophage-derived eCIRP release, which, in turn, mediates ZBP1-dependent PVEC PANoptosis in sepsis-induced ALI. This finding offers new insights into the molecular mechanisms driving sepsis-related pulmonary complications and provides potential new therapeutic strategies.

**Supplementary Information:**

The online version contains supplementary material available at 10.1186/s40779-024-00574-z.

## Background

Sepsis is a potentially life-threatening syndrome characterized by organ failure, resulting from a dysregulated host response to infection, which leads to acute organ dysfunction [1]. Sepsis-related deaths account for 19.7% of all global deaths [2]. The lungs are considered the most vulnerable target organ in sepsis, often developing acute lung injury (ALI) early in the sepsis course, making it a major cause of death among septic patients [3]. A comprehensive understanding of the mechanism underlying ALI in sepsis is essential for creating effective treatment strategies.

Cold-inducible RNA-binding protein (CIRP) was first described in the late 1990s as an RNA chaperone controlling the expression of cell cycle proteins in hibernating animals [4]. Since then, intense research on CIRP has revealed its role in the regulation of various cellular stress responses [5, 6], stress adaptation [7], and inflammation [8, 9]. CIRP is constitutively expressed at low levels across multiple tissues [10, 11], and can be upregulated during shock and trauma [8, 9]. Elevated serum levels of CIRP have been observed in patients with septic shock and correlate with the severity of organ injury [8].

Extracellular CIRP (eCIRP) has been defined as a damage-associated molecular pattern (DAMP) and an inflammatory mediator, which induces a range of cellular responses, including the secretion of pro-inflammatory cytokines and endothelial dysfunction [12, 13]. In cells treated with lipopolysaccharide (LPS), CIRP translocates from the nucleus to the cytosol [14], subsequently being released to the extracellular space through the lysosomal and exosomes secretion [8, 15]. Exosomes are also involved in the occurrence and progression of sepsis [16]. However, the mechanism underlying CIRP regulation and translocation in sepsis remains to be fully addressed.

The death of pulmonary vascular endothelial cells (PVECs) directly contributes to the development of ALI following sepsis [17, 18]. Endothelial cells (ECs) are the first cells to interact directly with circulating eCIRP. It has been reported that intravenous administration of eCIRP induces systemic inflammation and ALI via EC activation [19]. Notably, absent in melanoma 2 (AIM2), pyrin, and Z-DNA binding protein 1 (ZBP1) have been identified as components of a large multi-protein complex that includes apoptosis-associated speck-like protein containing a CARD (ASC), caspase-1 (CASP1), caspase-8 (CASP8), receptor-interacting protein kinase 3 (RIPK3), receptor-interacting protein kinase 1 (RIPK1), and Fas-associated protein with death domain (FADD), driving a unique inflammatory cell death, named PANoptosis [20]. ZBP1 acts as an essential mediator of PANoptosis by activating RIPK3, CASP8, and the NOD-like receptor family pyrin domain containing 3 inflammasome [21].

This study aimed to elucidate the molecular mechanism underlying sepsis-induced PANoptosis in PVECs and the subsequent ALI. The aim of this study was to investigate the mechanism by which lactylation of CIRP in macrophages leads to the release of eCIRP, its subsequent internalization by PVECs through a TLR4-mediated endocytosis pathway, and how this interaction with ZBP1 influences ZBP1-RIPK3-dependent PANoptosis in PVECs, thereby contributing to ALI.

## Methods

### Recruitment of participants and collection of samples

Ethical approval for this study was granted by the Medical Ethics Committee of Shenzhen Hospital of Southern Medical University (NYSZYYEC20200039). The study has been registered with the Clinical Trials Registry under the identifier ChiCTR2100043761. Recruitment of healthy volunteers was conducted through hospital personnel and public announcements. The study encompassed patients admitted to the Intensive Care Unit (ICU) of Shenzhen hospital from November 2018 to December 2021. Diagnosis of sepsis and septic shock followed the criteria outlined in the Third International Consensus Definitions for Sepsis (Sepsis-3). Patients were eligible for inclusion in the study if they were over 18 years old and within 24 h after admission to the ICU. Exclusion criteria included pregnancy, immunosuppression, leukopenia, hematological malignancies, or initiation of palliative care.

### Animal strains

All animal experimental protocols were reviewed and approved by the Institutional Animal Care and Use Committees of the University of Pittsburgh (IS0002501524045015) and Veterans Affairs Pittsburgh Healthcare System (1,617,201). C57BL/6 wild type (WT) mice (*n* = 50) were purchased from the Jackson Laboratories. *Tlr4*^*−/−*^ mice (*n* = 10), *Tlr2*^*−/−*^ mice (*n* = 10), *Casp8*^*−/−*^ mice (*n* = 10), *Ripk3*^*−/−*^ mice (*n* = 20), and *Zbp1*^*−/−*^ mice (*n* = 40) were obtained from the University of Pittsburgh.

### Cecal ligation and puncture (CLP) model

The mice were intraperitoneal administration of 50 mg/kg ketamine and 5 mg/kg xylazine. The cecum was exteriorized, ligated with 4–0 silk sutures, and punctured once using a 22-gauge needle by one through-and-through puncture after a 1.5 cm abdominal incision. Subsequently, we used 4–0 sutures to close the incision and reintroduce the cecum into the peritoneal cavity. The mice were observed for mortality every 6 h during the survival experiments. Twenty-four hours post-CLP surgery, the mice were euthanized for various experiments, from which blood, bronchoalveolar lavage fluid (BALF), and lung tissues were collected.

### Mouse PVECs (MPVECs) isolation and culture

Mice were anesthetized by intraperitoneal injection of 50 mg/kg ketamine and 5 mg/kg xylazine, after which the chest cavity was opened. The right ventricle was cannulated to infuse PBS to remove blood from the lungs. Peripheral lung tissue was diced to approximately 1 mm^3^ pieces and cultured in a 60 mm culture dish containing growth medium [MEMD-Val medium (Invitrogen Gibco, USA) supplemented with 2 mmol/L glutamine, 10% fetal bovine serum (FBS), 5% human serum, 50 μg/ml penicillin/streptomycin, 5 μg/ml heparin, 1 μg/ml hydrocortisone, 80 μg/ml EC growth supplement from bovine brain, 5 μg/ml amphotericin, and 5 μg/ml mycoplasma removal agent] at 37 °C with 5% CO_2_ for 60 h. Following this period, the tissue dice were removed while allowing adherent cells to continue culturing for an additional 3 d. The mouse lung vascular endothelial cells (MLVECs) were purified from these cultured cells using biotin-conjugated rat anti-mouse CD31 (PECAM-1) monoclonal antibody along with BD IMag streptavidin particles plus-DM, as per the manufacturer’s protocol provided by BD Biosciences (USA). The purified MLVECs were allowed to grow for 3 to 4 d before being characterized based on their cobblestone morphology, uptake of Dil-Ac-LDL (Biomedical Technologies, USA), and staining for factor VIII-related Ag (Sigma-Aldrich, USA).

### Isolation and culture of alveolar macrophages

Alveolar macrophages were obtained through bronchoalveolar lavage. The collected lavage fluid was centrifuged at 300 × *g* for 10 min at 4 °C. The cell pellet was then resuspended in DMEM with 10% FBS and 50 μg/ml penicillin/streptomycin for culture. After a 2 h incubation, non-adherent cells were removed by washing with PBS, and the culture medium was subsequently refreshed.

### Isolation, characterization, labeling, and uptake of exosome

Exosomes were extracted using a modified differential ultracentrifugation protocol (Beckman Coulter, USA). The first stage involved the removal of larger cellular fragments or debris via centrifugation at 3000 × *g* for 10 min at 4 °C. The remaining smaller cellular debris in the supernatant was further eliminated by centrifuging at 10,000 × *g* for 30 min at 4 °C. The supernatant fluid was then filtered using a 0.22 m filter (Millipore, USA) followed by centrifugation at 100,000 × *g* for 120 min at 4 °C (Beckman 436C Optima X, Beckman Coulter, USA). For exosome purification, the pellets were washed thrice with 1 × PBS and centrifuged at 100,000 × *g* for 120 min at 4 °C. Exosome pellets were collected, resuspended in PBS, sub-packed, and stored at −80 ℃. Then, protein quantification was conducted using a bicinchoninic acid assay kit (Pierce, USA).

### Cell culture and isolation of peritoneal macrophages

Human umbilical vein endothelial cells (HUVECs) and mouse alveolar macrophages (MH-S) cells were obtained from ATCC (Manassas, Virginia, USA). The cells were cultured in DMEM (Invitrogen, Grand Island, New York, USA), respectively. All cultured media were supplemented with 10% heat-inactivated FBS, 1% penicillin/streptomycin, and 2 mmol/L glutamine. Cells were maintained in a 37 °C incubator with 5% CO_2_.

### Administration of recombinant mouse CIRP (rmCIRP)

Mouse CIRP/CIRBP protein (recombinant 6His, N-terminus, full length) was obtained from LifeSpan BioSciences, Inc. (Catalog number: LS-G136982). Mice were divided into 2 groups: vehicle and rmCIRP. A small incision was made on the neck to access the internal jugular vein. Normal saline (vehicle) or rmCIRP at a dosage of 5 mg/kg in a volume of 200 μl was injected into the jugular vein using a 29G × 1/2″ U-100 insulin syringe (Terumo Medical Corporation, USA).

### Apoptosis analysis

Following the protocols of an Annexin V-FITC/PI Cell Apoptosis Detection Kit (BD Biosciences, USA), Annexin V-FITC binding solution and propidium iodide (PI) were added. Data analysis was performed using FlowJo software (version 10.0.7, Tree Star, Inc., USA).

### Western blotting

Proteins were extracted using RIPA lysis buffer supplemented with protease inhibitors (Sigma, USA). The concentration of the extracted protein was quantified using a BCA protein assay kit (Thermo, USA). Equal amounts of total proteins (30 μg) were separated by 10% SDS-PAGE and subsequently transferred to a PVDF membrane (Merck Millipore, Germany). The membrane was blocked with 5% non-fat milk at room temperature for 1 h and incubated overnight at 4 ℃ with specific primary antibodies. After incubation with horseradish peroxidase-conjugated secondary antibodies, the protein bands were visualized using enhanced chemiluminescence reagents (Merck Millipore, Germany). Primary antibodies, including CIRP, protein kinase R (PKR)-like endoplasmic reticulum kinase (PERK), phosphorylated PKR-like endoplasmic reticulum kinase (p-PERK), eukaryotic initiation factor 2 alpha (eIF2α), phosphorylated eukaryotic initiation factor 2 alpha (p-eIF2α), activating transcription factor 4 (ATF4), C/EBP homologous protein (CHOP), CASP1, gasdermin D (GSDMD), CASP8, CASP3, RIPK1, RIPK3, mixed lineage kinase domain-like protein (MLKL), ZBP1, β-actin, and GAPDH, are listed in Additional file 1: Table S1. Images were captured using a ChemiDoc imaging system (Bio-Rad, USA).

### Immunoprecipitation (IP) and immunoblotting

Cells were lysed using IP lysis buffer (Thermo, USA) and kept on ice with continuous mixing for 30 min. Following centrifugation at 12,000 × *g* for 15 min, 500 μl of the supernatant (containing 500 μg of protein) was precleared by incubating with 20 μl of a 50% slurry of protein A/G magnetic beads (Merck Millipore, Germany) at 4 °C for 1 h. Utilizing a magnetic stand, the beads were separated, and the supernatant was subsequently incubated with 4 μg of antibodies (refer to Additional file 1: Table S1) specific to the target protein at 4 °C overnight. The next day, an additional 50 μl of protein A/G magnetic beads were added to the solution and incubated on a rotary shaker at 4 °C for 2 h. The beads were then collected and washed 6 times with 1 ml PBS. The bound proteins were eluted and analyzed via SDS-PAGE before being transferred to Immobilon-P membranes for Western blotting.

### Real-time cell death analysis

Real-time cell death assays were performed using an IncuCyte S3 imaging system (Essen Biosciences, USA). Cells were seeded in 12-well plates at a density of 10^6^ cells/well and subsequently treated with the specified cytokines, followed by staining with PI (Life Technologies, USA, P3566) according to the manufacturer’s protocol. The plate was scanned, capturing fluorescent and phase-contrast images (4 image fields per well) in real-time every 1 h from 0 to 48 h post-treatment. PI-positive dead cells were marked with a red mask for enhanced visualization. The image analysis, masking, and quantification of dead cells were performed using the software package supplied with the In-cuCyte imager.

### Immunofluorescence staining

Cells or tissues were fixed in 4% paraformaldehyde at room temperature for 30 min, permeabilized with 0.1% Triton X-100 for 5 min, and then blocked with 5% bovine serum albumin (BSA) at room temperature for another 30 min. Primary antibodies targeting CIRP, TLR4, early endosome antigen 1 (EEA1), CD31, F4/80, lactylated lysine (Klac), ZBP1, and others (refer to Additional file 1: Table S1) were incubated overnight at 4 °C. The following day, slides were treated with fluorescently labeled secondary antibodies diluted to a ratio of 1:200 for 1 h in the dark. Cell nuclei were stained with DAPI for 5 min. Samples were then visualized and imaged using a Nikon A1R confocal microscope.

### Proximity ligation assay (PLA)

For PLA, cells were cultured in confocal dishes, washed with PBS, and fixed with 4% formaldehyde for 15 min. Blocking was conducted with 5% BSA for 30 min, followed by overnight incubation at 4 °C with antibodies targeting ZBP1 and TRIM32 or RIPK3. The subsequent steps of probe incubation, ligation, and amplification were carried out according to the manufacturer’s protocol provided in the Duolink Detection Kit (DUO92102-1KT, Sigma-Aldrich, USA). Nuclei were stained with DAPI, and slides were mounted. PLA samples were imaged and analyzed using a 60 × objective on a Nikon A1R confocal microscope.

### Single-cell suspension preparation, library construction, and sequencing

Mice were euthanized 24 h post-surgery, and lung tissues were promptly excised. Under sterile conditions, tissues were washed twice with ice-cold PBS supplemented with 0.04% BSA. Clean tissues were minced into approximately 0.5 mm^3^ fragments using sterile surgical scissors and placed in freshly prepared digestion solution. Digestion was performed at 37 °C for 30 min with intermittent stirring every 10 min. The resulting cell suspension was filtered through a 70 μm cell strainer (Becton Dickinson, USA) twice, followed by centrifugation at 400 × *g* for 5 min at 4 °C. The pellet was resuspended in medium, and mixed with an equal volume of red blood cell lysis buffer before being incubated at 4 °C for an additional 5 min. After another round of centrifugation at 400 × *g* for 5 min, the supernatant was discarded. The pellet was washed once with medium, subjected to further centrifugation, and then resuspended in 100 μl of medium. The single-cell suspension concentration was adjusted to a density of 30,000 cells per sample. Library construction followed the manufacturer’s instructions for the 10 × Genomics Chromium Next GEM Single Cell 3’ Reagent Kit v3.1 (Catalog number: 000388). The libraries were sequenced on the NovaSeq 6000 platform using high-throughput sequencing technology.

### Hematoxylin and eosin (H&E) staining and lung injury score

The lungs were fixed in 4% paraformaldehyde. After fixation, the lungs were embedded in paraffin, cut into 5 μm sections, and subjected to H&E staining according to routine histopathological methods. Histopathological changes were observed under a light microscope. The degree of lung injury (atelectasis, alveolar and interstitial inflammation, alveolar and interstitial hemorrhage, alveolar and interstitial edema, necrosis, and overdistension) was evaluated in 6 sections from the lower lobes using the following criteria: 0 indicating no injury; 1 for injury to 25% of the field; 2 for injury to 50% of the field; 3 for injury to 75% of the field; 4 representing diffuse injury. Lung injury was evaluated by independent pathologists who were blinded to the group allocation.

### Luciferase assay and site-directed mutagenesis

Luciferase constructs driven by the ATF4 promoter were generated using specific primers (refer to Additional file 1: Table S2). The 293 T cell line (American Type Culture Collection, USA) was cultured in 24-well plates before transfection with CIRP vectors and promoter constructs using Lipofectamine 2000. The Dual-Luciferase assay system was then employed for the analysis of reporter gene expression.

### Statistical analysis

All data analyses were performed using GraphPad Prism software version 8.0. The data are presented as the mean ± SD from at least 3 independent experiments, or as median (IQR) for descriptive data. Survival curves were constructed employing the Kaplan–Meier method and subsequently analyzed using the log-rank test. Differences were considered statistically significant at *P* < 0.05. The results were analyzed using Student’s *t*-test for two groups and one-way ANOVA analysis for multiple groups.

## Results

### Elevated exosomal CIRP in plasma and BALF correlates with lactate levels and severity of *sepsis*-induced ALI

**Fig. 1:**
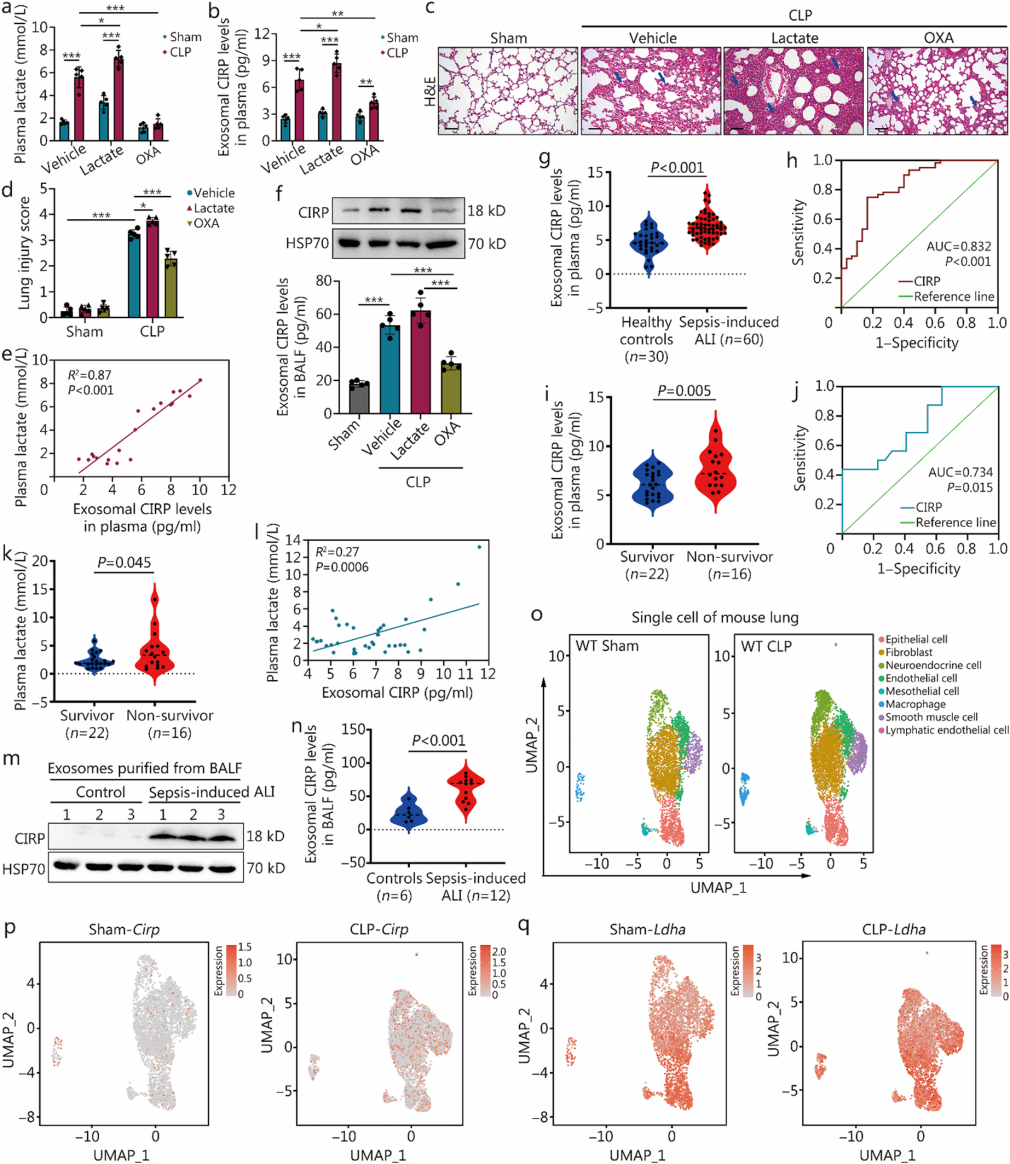
Elevated exosomal cold-inducible RNA-binding protein (CIRP) in plasma and bronchoalveolar lavage fluid (BALF) correlates with lactate levels and severity of sepsis-induced acute lung injury (ALI). **a-f** Mice were administered lactate (0.5 g/kg) intraperitoneally 6 h after cecal ligation and puncture (CLP) or sham. To inhibit lactate production, sodium oxamate (OXA, 0.5 g/kg) was injected intraperitoneally 6 h before CLP or sham. Mice were euthanized 24 h post-CLP. Mouse plasma lactate levels were measured using a lactate assay kit (**a**, *n* = 5). Plasma exosomal CIRP levels in mice were detected using an enzyme-linked immunosorbent assay (ELISA) kit across groups (**b**). Hematoxylin and eosin (H&E) staining of lung tissues from sham and CLP mice, with arrows indicating lung injury characterized by alveolar septal thickening and inflammatory cell infiltration (**c,** scale bar = 50 μm). Lung injury scores were statistically evaluated for each group (**d**). Significant correlations were analyzed between blood lactate levels and plasma exosomal CIRP levels across groups (**e**). Plasma exosomal CIRP levels were measured by Western blotting and ELISA among sham, CLP, CLP + lactate, and CLP + OXA groups (**f,** *n* = 5). **g** ELISA analysis of clinical samples demonstrates significantly higher levels of plasma exosomal CIRP in patients with sepsis-induced ALI compared to healthy controls. **h** ROC curve analysis indicates that exosomal CIRP has high diagnostic and prognostic value for sepsis-induced ALI. **i** ELISA detection shows that plasma exosomal CIRP levels are higher in non-survivors than survivors among patients with sepsis. **j** ROC curve for exosomal CIRP levels predicting patient outcomes in sepsis with AUC = 0.734. **k** Plasma lactate levels are significantly elevated in non-survivors compared to survivors in sepsis. **l** Correlation analysis reveals a significant positive relationship between plasma exosomal CIRP levels and plasma lactate levels. **m** Western blotting analysis indicates significantly increased exosomal CIRP levels in the BALF of patients with sepsis-induced ALI compared to controls. **n** ELISA analysis confirms significantly higher exosomal CIRP levels in the BALF of a larger cohort of patients with sepsis-induced ALI compared to controls. **o** Single-cell RNA sequencing was performed on the lungs of sham and CLP mice at 24 h (*n* = 3). Cell populations were identified based on copy number variations inferred from expression (annotations for each cell population are provided in the Additional file 1: Fig. S1). Cells colored by the expression levels of *Cirp* (**p**) and *Ldha* (**q**) in the lungs of mice. Data are represented as mean ± SD. ^*^P < 0.05*,* ^**^P < 0.01*,* ^***^P < 0.001. ROC receiver operating characteristic, AUC area under the curve, Ldha lactate dehydrogenase A

Using the CLP sepsis mouse model, we observed significant increases in plasma lactate (Fig. 1a) and exosomal CIRP (Fig. 1b) in CLP mice compared to that in sham controls. To explore the regulatory role of lactate on exosomal CIRP production, we intraperitoneally (i.p.) injected lactate to the mice at 6 h post-CLP to elevate serum lactate levels. As depicted in Fig. 1a, b, at 24 h after CLP, lactate administration led to increased plasma lactate and exosomal CIRP levels, as well as a more pronounced exacerbation of lung injury (Fig. 1c, d) compared to controls. Conversely, sodium oxamate (OXA), a specific inhibitor of lactate dehydrogenase suppressing lactate production, significantly attenuated the sepsis-induced increases in plasma lactate (Fig. 1a) and exosomal CIRP (Fig. 1b), while also ameliorating lung injury in septic mice (Fig. 1c, d). Notably, the sham group displayed normal lung histology, whereas the vehicle- and lactate-treated CLP groups exhibited significant lung injury characterized by inflammation, edema, and tissue damage. The OXA treatment appeared to mitigate these pathological changes (Fig. 1c, d). Further correlation analysis confirmed a positive relationship between exosomal CIRP and plasma lactate levels in all mice, including both the CLP and sham groups (Fig. 1e). Additional experiments involving the extraction of exosomes from the BALF of mice and measuring exosomal CIRP levels further supported the finding that inhibiting lactate production could significantly decrease the release of exosomal CIRP induced by CLP (Fig. 1f).

These preclinical findings were also observed in real-world clinical studies. We identified a significant correlation between the levels of exosomal CIRP in plasma and BALF and lactate levels, and the severity of post-sepsis ALI. Exosomes were isolated from the plasma of both patients with sepsis-induced ALI (*n* = 60) and healthy controls (*n* = 30), as detailed in Additional file 1: Table S3. The concentration of plasma exosomal CIRP was quantified using enzyme-linked immunosorbent assay (ELISA), revealing a notable elevation in the sepsis-induced ALI patient group compared to healthy controls (Fig. 1g). Receiver operating characteristic (ROC) curve analysis yielded an area under the curve (AUC) of 0.832 for exosomal CIRP (Fig. 1h), indicating its potential as a robust diagnostic marker for sepsis-induced ALI.

We further classified patients into survival (*n* = 22) and non-survival (*n* = 16) groups based on the outcomes documented in Additional file 1: Table S4. Notably, the non-survival group exhibited higher levels of exosomal CIRP than the survival group (Fig. 1i). The prognostic accuracy of exosomal CIRP was evidenced by an AUC of 0.734 on the ROC curve (Fig. 1j), demonstrating meaningful sensitivity and specificity in predicting patient outcomes. Thus, the elevated levels of plasma exosomal CIRP are strongly associated with sepsis-induced ALI and correlate with adverse patient prognosis.

Clinical studies have established that high serum lactate levels are associated with increased mortality rates in patients with sepsis [22]. Our findings align with this, demonstrating that lactate levels are significantly elevated in the non-survival group compared to the survival group among sepsis patients (Fig. 1k). Additionally, we identified a significant positive correlation between plasma exosomal CIRP and lactate levels (Fig. 1l), underscoring the potential of exosomal CIRP as a biomarker for sepsis severity.

To further investigate the connection between exosomal CIRP and lung injury, we isolated exosomes from the BALF of patients with sepsis-induced ALI. In comparison to a control group of patients who underwent bronchoscopy without sepsis (*n* = 3), the level of exosomal CIRP in BALF was significantly higher in the sepsis-induced ALI group (Fig. 1m). We expanded our analysis to a larger cohort, contrasting sepsis-induced ALI patients (*n* = 12) with non-septic ALI controls (*n* = 6), and observed increased levels of exosomal CIRP in the BALF of the sepsis-induced ALI group (Fig. 1n). These data indicate a critical link between exosomal CIRP and lung injury in sepsis.

To elucidate the origin of exosomal CIRP, we conducted single-cell RNA sequencing on lung tissues from sepsis and sham-operated mice (*n* = 3). Using uniform manifold approximation and projection (UMAP), we identified cell clusters based on the expression profiles of established cell type-specific marker genes (Fig. 1o; Additional file 1: Fig. S1a). The single-cell RNA sequencing analysis showed distinct cellular clustering within the lung tissue, and upregulation of both *Cirp* and *Ldha* expressions can be detected across various cells in the lungs of CLP mice (Fig. 1p, q), while macrophage populations exhibited a predominant expression of CIRP (Additional file 1: Fig. S1b, c).

### Lactylation of ATF4-upregulated CIRP in macrophages promotes CIRP release in *sepsis*

**Fig. 2:**
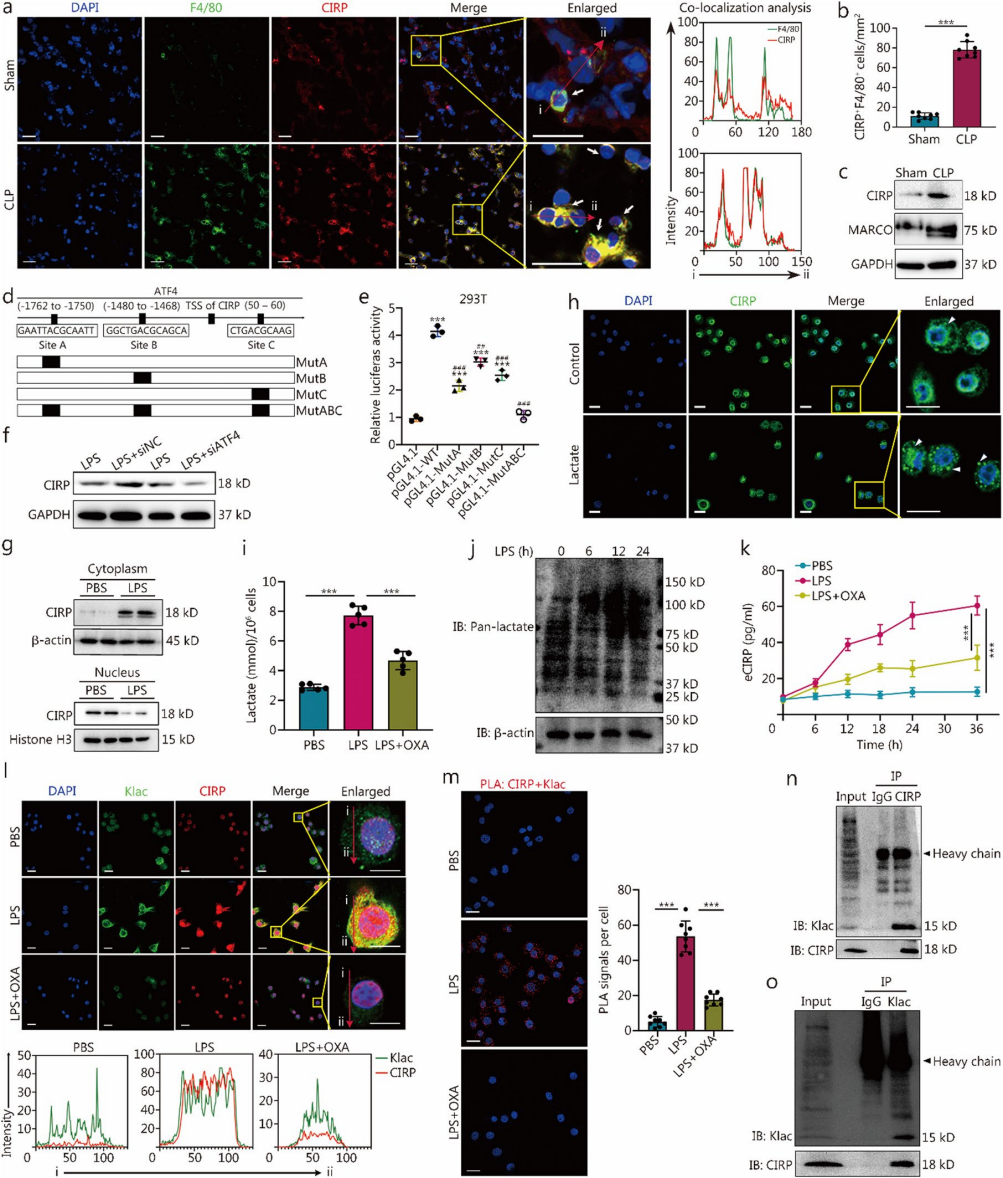
Lactylation of activating transcription factor 4 (ATF4)-upregulated cold-inducible RNA-binding protein (CIRP) in macrophages promotes CIRP release in sepsis. **a** Immunofluorescence shows significantly increased expression of CIRP (red) in F4/80 (green) macrophages in the lung tissue of cecal ligation and puncture (CLP) mice, with the right panel showing colocalization analysis (scale bar = 20 µm). The arrows from “i” to “ii” highlight the region analyzed for co-localization analysis. **b** Quantitative analysis indicates a higher number of CIRP^+^F4/80^+^ cells in the CLP group compared to the sham. **c** Western blotting analysis of bronchoalveolar lavage fluid (BALF) macrophages from CLP mice shows a significant increase in CIRP expression. **d** Promoter sequences of *Cirp* with 3 binding sites for ATF4 were predicted using databases such as University of California Santa Cruz (UCSC) and JASPAR (wild type), and mutant binding site constructs (MutA, MutB, MutC, and MutABC) were generated. **e** Dual-luciferase assays demonstrate ATF4 binding to the *Cirp* promoter region and transcriptional regulation of *Cirp* expression. **f** Western blotting analysis shows a significant increase in CIRP expression following LPS stimulation in cells, which is notably reduced after ATF4 interference. **g** Western blotting analysis was performed to determine cytoplasmic and nuclear CIRP levels in mouse alveolar macrophage (MH-S) cells after 6 h of stimulation with 1 µg/ml LPS. **h** Representative immunofluorescence images of MH-S cells treated with either vehicle or lactate (10 mmol/L) for 6 h. Lactate-treated cells exhibit increased cytoplasmic accumulation of CIRP (indicated by white arrows) (scale bar = 20 µm). **i** Intracellular lactate levels were measured using a kit after 6 h of treatment with LPS or LPS + OXA. **j** Pan-lactylation levels in macrophages following LPS stimulation (at 0, 6, 12, and 24 h) were assessed using Western blotting analysis. **k** The content of CIRP in the cell supernatant of alveolar macrophages stimulated with LPS or LPS + OXA was measured by enzyme-linked immunosorbent assay (ELISA), with PBS serving as the control group. **l** MH-S cells were treated with LPS or LPS + OXA for 24 h (scale bar = 20 µm). Confocal microscopy was used to examine the colocalization of CIRP (red) and Klac (green). DAPI (blue) staining indicates the nuclei. Below are colocalization analyses using ImageJ. The arrows from “i” to “ii” highlight the region analyzed for co-localization analysis. **m** A proximity ligation assay (PLA) using specific antibodies against CIRP and lactylated lysine (Klac), with nuclei stained using DAPI (scale bar = 20 μm). The right panel is a statistical graph of PLA. After 24 h of LPS stimulation in macrophages, cell lysates precipitated with agarose beads underwent a pull-down assay and were probed for CIRP and Klac protein levels using either control IgG or CIRP monoclonal antibody (**n**) and Klac monoclonal antibody (**o**). * Indicates comparison with the pGL4.1 control group, and ^#^ indicates comparison with the pGL4.1-WT group. Data are presented as the mean ± SD. ^***^P < 0.001; ^##^*P* < 0.01, ^###^*P* < 0.001. OXA sodium oxamate, MARCO macrophage receptor with collagenous structure, LPS lipopolysaccharide, DAPI 4',6-diamidino-2-phenylindole, IB immunoblotting

Histological analysis of lung tissue post-sepsis showed a marked increase in alveolar macrophage infiltration, accompanied by a significant elevation in CIRP expression observed at 24 h (Fig. 2a, b). Western blotting revealed that following CLP, CIRP expression in macrophages from BALF was significantly higher compared to the sham group (Fig. 2c).

Next, we observed a notable upregulation of endoplasmic reticulum (ER) stress-related proteins in the lungs of septic mice, including p-PERK, p-eIF2α, ATF4, and CHOP (Additional file 1: Fig. S2a). These findings were consistent with the ER swelling and vacuolation seen in macrophages following LPS challenge (Additional file 1: Fig. S2b). Using bioinformatic tools from the University of California, Santa Cruz (UCSC) [23], PROMO [24], and JASPAR [25], we identified 3 putative ATF4-binding motifs within the *Cirp* promoter region, labeled as sites A, B, and C (Fig. 2d). Through luciferase reporter assays, we confirmed that ATF4 directly binds to these sites and enhances the activity of the *Cirp* promoter. Individual mutations introduced at these loci exhibited a diminished effect, whereas a triple mutation (sites A, B, and C combined) abrogated the ATF4-mediated transcriptional activation of *Cirp* (Fig. 2e). Furthermore, we performed chromatin IP followed by qPCR to monitor ATF4 binding at the *Cirp* promoter, revealing its role in transcriptional regulation of *Cirp* expression (Additional file 1: Fig. S2**c**). Knockdown experiments further delineated the pivotal role of ATF4 in modulating CIRP levels in response to LPS stimulation (Fig. 2f).

Moreover, we found that LPS induced the translocation of CIRP from the nucleus to the cytoplasm in macrophages (Fig. 2g), and this effect was also observed when macrophages were treated with 10 mmol/L lactate [22, 26] (Fig. 2h), suggesting that cytoplasmic CIRP is concentrated and committed to the exosome pathway for subsequent release [8] (Additional file 1: Fig. S3).

A recent study has demonstrated that lactate promotes the lactylation of high mobility group box 1 (HMGB1) and its subsequent secretion via exosomes in macrophages [27]. We treated macrophages with LPS in the presence or absence of OXA and examined lactylation levels in cell extracts. The results indicated that LPS increases lactate production (Fig. 2i) and significantly elevates the levels of pan-lactylation proteins in macrophages (Fig. 2j).

As depicted in Fig. 2k, LPS significantly increased the level of eCIRP in the cell supernatant, an effect that was reversed by OXA. To determine if lactate serves as the mediator for LPS-induced CIRP lactylation, we inhibited endogenous lactate production using OXA before LPS stimulation. This intervention strongly diminished the lactylation of CIRP induced by LPS in macrophages (Fig. 2k). Immunofluorescence staining showed that LPS promotes the colocalization of Klac and CIRP within the cytoplasm of alveolar macrophages (Fig. 2l). To validate the direct interaction and lactylation of CIRP by Klac, we performed a PLA, which yields a positive signal (red immunofluorescence dots) only when Klac is close to CIRP. The results revealed a direct interaction between Klac and CIRP, whereas OXA notably reduced LPS-induced CIRP lactylation in macrophages (Fig. 2m). IP data demonstrated that LPS significantly increased Klac levels within the CIRP immune complexes (Fig. 2n), and high levels of CIRP expression were also detected in the Klac immune complexes following LPS stimulation (Fig. 2o).

In summary, sepsis induces an accumulation of lactate within macrophages, leading to the lactylation of CIRP. This post-translational modification promotes the translocation of CIRP from the nucleus to the cytoplasm and its subsequent release.

### TLR4 mediates the endocytosis of eCIRP in MPVECs

**Fig. 3:**
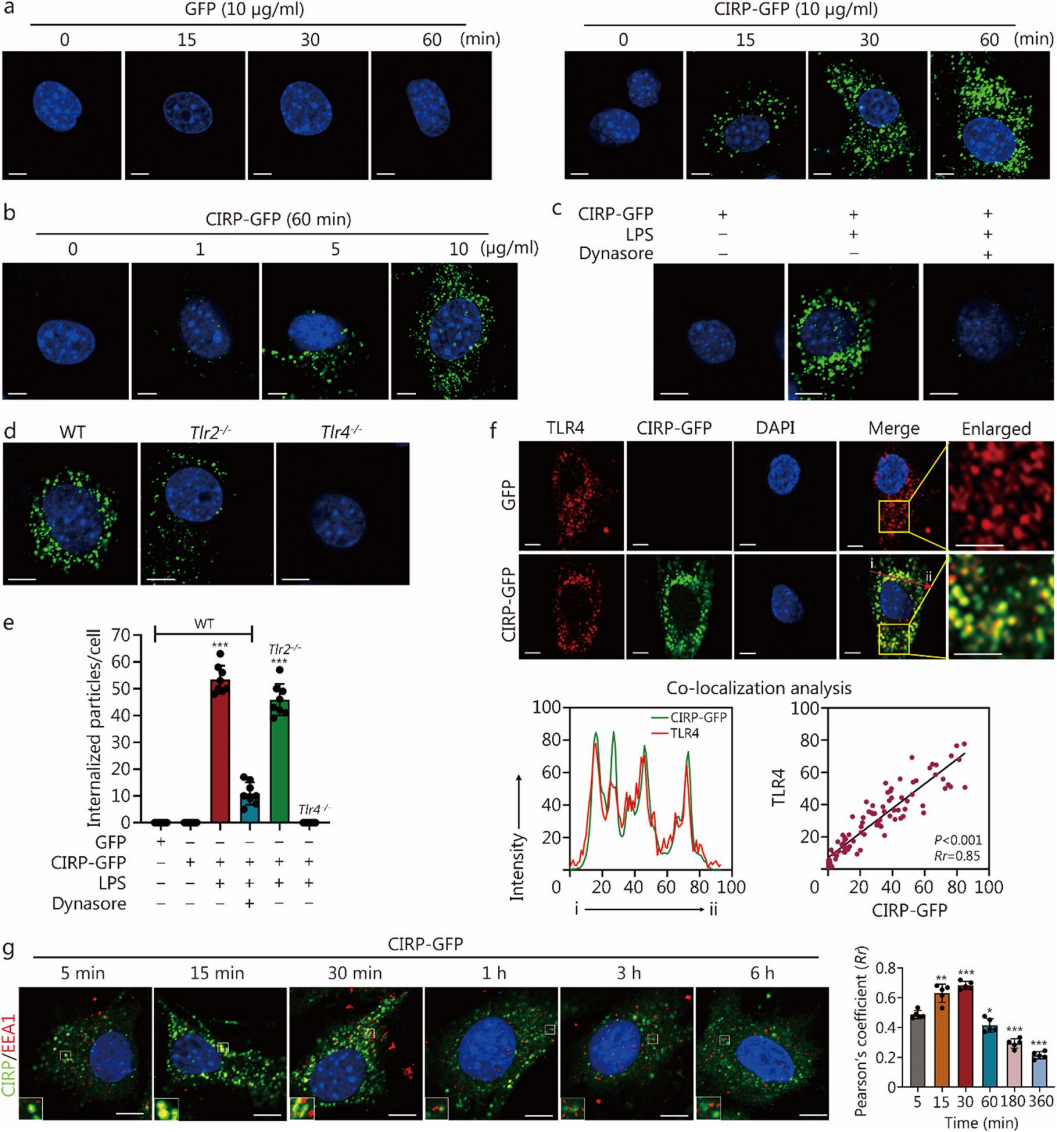
Toll-like receptor 4 (TLR4) mediates the endocytosis of eCIRP in mouse pulmonary vascular endothelial cells (MPVECs). **a** Confocal microscopy images of MPVECs isolated from C57BL/6 [wild type (WT)] mice, incubated with recombinant CIRP-GFP (10 µg/ml) or green fluorescent protein (GFP) in the presence of LPS (25 µg/ml) for 0 − 60 min (scale bar = 5 µm). **b** Confocal microscopy images of MPVECs isolated from WT mice, incubated with 0 − 10 µg/ml CIRP-GFP in the presence of LPS (25 µg/ml) for 30 min (scale bar = 5 µm). **c** Confocal microscopy images of WT MPVECs incubated with CIRP-GFP in the presence or absence of dynasore (30 µg/ml) or LPS (25 µg/ml) for 30 min (scale bar = 5 µm). **d** Confocal microscopy images of MPVECs isolated from WT, *Tlr2*^*−/−*^, or *Tlr4*^*−/−*^ mice, incubated with CIRP-GFP (10 µg/ml) in the presence of LPS (25 µg/ml) for 30 min (scale bar = 5 µm). **e** Quantification of the average number of intracellular GFP-positive particles in MPVECs, calculated using a confocal microscopy program. MPVECs were isolated from WT, *Tlr2*^*−/−*^, or *Tlr4*^*−/−*^ mice and incubated with CIRP-GFP (10 µg/ml) for 30 min. The first column serves as the control group, and the symbols *, **, and *** represent statistically significant differences with *P*-values compared to the control group. **f** Confocal microscopy images showing co-localization of CIRP-GFP and TLR4 in MPVECs isolated from WT mice, incubated with CIRP-GFP and LPS for 30 min (scale bar = 5 µm). Line graphs below the images display co-localization analysis of CIRP-GFP and TLR4. The arrow from “i” to “ii” highlights the region analyzed for co-localization analysis. **g** Immunofluorescence detection of early endosome antigen 1 (EEA1, red) in WT MPVECs treated with CIRP-GFP (green, 10 µg/ml) for 5, 15, 30 min, and 1, 3, or 6 h (scale bar = 5 µm). The bar chart shows statistical analysis of CIRP-GFP co-localization with EEA1. The first column serves as the control group, and the symbols *, **, and *** represent statistically significant differences with *P*-values compared to the control group. The data are presented as the mean ± SD. ^*^P < 0.05, ^**^P < 0.01, ^***^P < 0.001. eCIRP extracellular cold-inducible RNA-binding protein

CIRP, an endogenous DAMP molecule, is primarily recognized for its role as an inflammatory amplifier by activating signaling pathways through receptor binding [8]. However, we observed a rapid endocytosis of CIRP in MPVECs upon exposure to LPS. Treatment of MPVECs with CIRP conjugated to a green fluorescent protein (GFP, 10 µg/ml) induced the internalization of CIRP-GFP within just 5 min post-treatment. This internalization of CIRP-GFP represents a specific phagocytic action mediated by CIRP itself, as GFP alone did not exhibit any internalization (Fig. 3a). Notably, a significant endocytosis of CIRP-GFP was observed at a concentration of 10 µg/ml (Fig. 3b).

Previous research has identified TLR4 as the receptor for CIRP [8]. LPS can induce the internalization of TLR4 [28]. To further elucidate the mechanism underlying CIRP internalization, we observed that CIRP endocytosis is mediated by LPS. Treating cells with the dynamin inhibitor dynasore effectively blocked CIRP internalization, thereby confirming that its transport requires a dynamin-dependent pathway (Fig. 3c).

To ascertain whether CIRP-GFP internalization is a receptor-dependent process, we isolated MPVECs from *Tlr2*^*−/−*^ and *Tlr4*^*−/−*^ mice and subsequently treated them with CIRP-GFP. The results demonstrated that the deficiency of *Tlr4* prevented CIRP internalization, whereas the absence of *Tlr2* did not affect it (Fig. 3d, e), suggesting that CIRP internalization is dependent on TLR4 in the presence of LPS. LPS binding protein and CD14 facilitate the recognition and binding of LPS to TLR4, forming the LPS-TLR4-myeloid differentiation factor 2 (MD2) complex that initiates endocytosis [29]. This complex, upon binding LPS, triggers intracellular signaling pathways through adaptor proteins such as myeloid differentiation primary response 88 (MyD88) and TIR-domain-containing adapter-inducing interferon-β (TRIF), leading to endocytosis [30]. We also observed a significant co-localization of CIRP-GFP with TLR4 during the endocytosis process (Fig. 3f), further supporting the essential role of TLR4 in the intracellular transport of CIRP.

To track the translocation of internalized CIRP-GFP within MPVECs, we noted a significant co-localization between EEA1 and CIRP within 1 h post-LPS treatment (Fig. 3g). The Pearson’s coefficient between CIRP and EEA1 decreased after 60 min, indicating that CIRP is trafficked from early endosomes to other intracellular compartments over time (Fig. 3g).

In conclusion, these findings suggest that during sepsis, TLR4 mediates the endocytosis of CIRP, thereby initiating this process in MPVECs through a dynamin-dependent pathway.

### LPS and eCIRP induce MPVEC PANoptosis

To investigate the mechanism underlying MPVEC death, we assessed the roles of LPS and CIRP in the activation of the PANoptosis pathway, an integrated program of cell death that includes pyroptosis, apoptosis, and necroptosis.

**Fig. 4:**
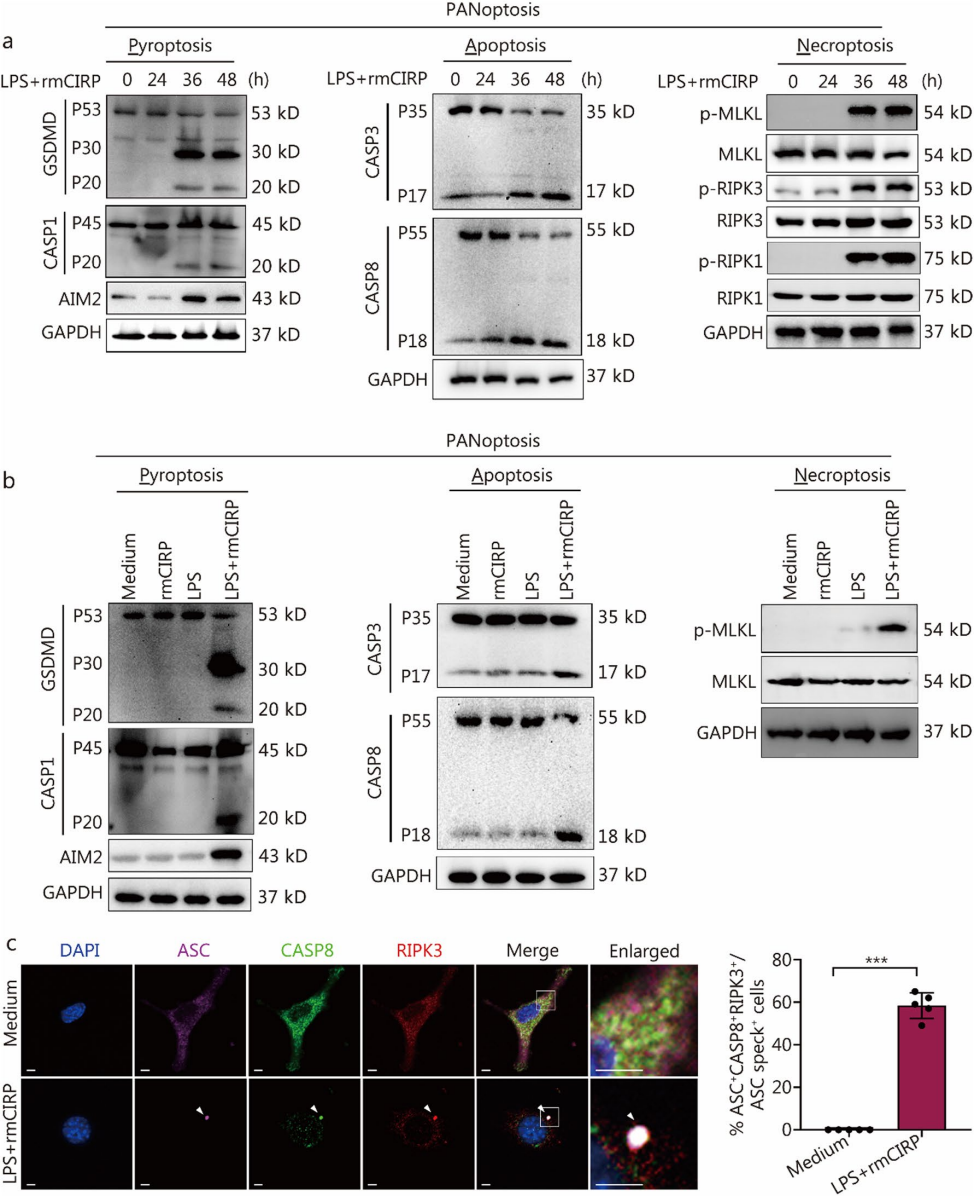
Lipopolysaccharide (LPS) and rmCIRP induce MPVEC PANoptosis. **a** Immunoblot analyses were conducted to evaluate the expression and activation of key cell death mediators. Pro-activated (P53), activated (P30), and inactivated (P20) GSDMD, pro-activated (P45) and activated (P20) CASP1, and activated AIM2; pro-cleaved (P35) and cleaved (P17) CASP3, pro-cleaved (P55) and cleaved (P18) CASP8; p-MLKL, MLKL, p-RIPK3, RIPK3, p-RIPK1, and RIPK1 in primary MPVECs co-treated with LPS and rmCIRP (10 µg/ml). **b** Immunoblot analysis of CASP1, GSDMD, and AIM2, CASP3 and CASP8, p-MLKL and MLKL in primary MPVECs treated with either LPS alone, rmCIRP alone, or co-treatment with LPS and rmCIRP for 48 h. **c** Immunofluorescence microscopy revealed the cellular localization of ASC specks in primary MPVECs 48 h co-treatment with LPS and rmCIRP (scale bar = 5 μm). Arrowheads indicate the PANoptosome. The bar chart presents the quantification of the percentage of cells with ASC^+^CASP8^+^RIPK3^+^ specks among the ASC speck^+^ cells. The data are expressed as the mean ± SD. ^***^P < 0.001. rmCIRP recombinant mouse cold-inducible RNA-binding protein, MPVEC mouse pulmonary vascular endothelial cell, GSDMD gasdermin D, CASP1 caspase-1, AIM2 absent in melanoma 2, CASP3 caspase-3, CASP8 caspase-8, p-MLKL phosphorylated mixed lineage kinase domain-like protein, MLKL total mixed lineage kinase domain-like protein, p-RIPK3 phosphorylated receptor-interacting protein kinase 3, RIPK3 receptor-interacting protein kinase 3, p-RIPK1 phosphorylated receptor-interacting protein kinase 1, RIPK1 receptor-interacting protein kinase 1, ASC apoptosis-associated speck-like protein containing a CARD

MPVECs treated with LPS and rmCIRP exhibited multiple forms of cell death, including pyroptosis, apoptosis, and necroptosis. Pyroptosis is evidenced by the presence of cleaved GSDMD fragments, P30 and P20, along with the activation of CASP1 indicated by its P20 form and an increase in AIM2 levels (Fig. 4a). Additionally, there was an elevation in IL-1β secretion and lactate dehydrogenase release (Additional file 1: Fig. S4). Apoptosis was characterized by the cleavage of CASP3 to its P17 fragment and CASP8 to its P18 form (Fig. 4a). Necroptosis was implicated by the phosphorylation of MLKL as well as RIPK3 and RIPK1 (Fig. 4a).

A comparison analysis of the individual and synergistic effects of LPS and rmCIRP treatments showed that the co-treatment significantly amplified the activation of pyroptosis and apoptosis (Fig. 4b), and also increased MLKL phosphorylation at 48 h after co-treatment (Fig. 4b). At 48 h following LPS and rmCIRP co-treatment, we noted the co-localization of ASC specks with CASP8 and RIPK3 within the same cells, forming a complex known as the PANoptosome (Fig. 4c). These results support the role of eCIRP in mediating PVEC PANoptosis.

### ZBP1 regulates PVEC PANoptosis in response to LPS and rmCIRP

ZBP1 has been identified as a key sensor of cytoplasmic double-stranded DNA and a mediator of inflammasome activation, leading to the induction of PANoptosis [31]. Recent studies have highlighted ZBP1’s involvement in mediating cellular death responses to diverse stress signals, including viral infections and inflammatory stimuli [21, 32]. Given its pivotal role in these processes, we hypothesized that ZBP1 might also play a significant role in PANoptosis of PVECs in response to LPS and eCIRP during sepsis-induced inflammation.

**Fig. 5:**
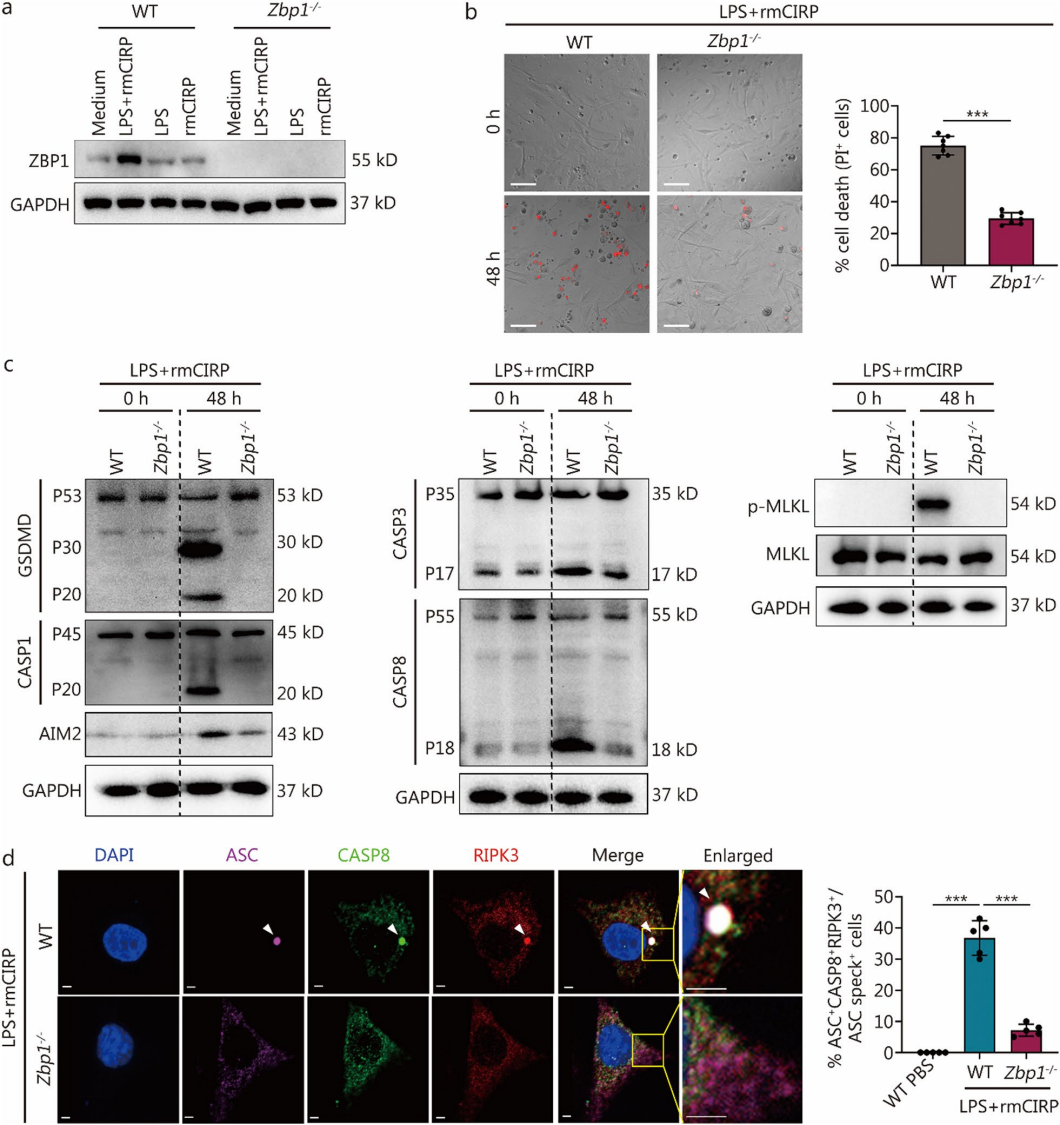
Z-DNA binding protein 1 (ZBP1) regulates pulmonary vascular endothelial cell (PVEC) PANoptosis in response to lipopolysaccharide (LPS) and extracellular CIRP (eCIRP). **a** Immunoblot analysis of ZBP1 in WT and *Zbp1*^*−/−*^ primary MPVECs after treatment with LPS alone, rmCIRP alone, or co-treatment with LPS and rmCIRP for 48 h. **b** Representative images of cell death and quantification showing the percentage of cell death in WT, *Zbp*^*−/−*^ primary MPVECs treated with LPS and rmCIRP for 48 h (scale bar = 100 μm).** c** Immunoblot analysis of pro-activated (P45) and activated (P20) CASP1; pro-activated (P53), activated (P30), and inactivated (P20) GSDMD; and AIM2; pro-cleaved (P35) and cleaved (P17) CASP3, pro-cleaved (P55) and cleaved (P18) CASP8; p-MLKL and MLKL in WT and *Zbp1*^*−/−*^ MPVECs at 0 h or co-treatment with LPS and rmCIRP for 48 h. **d** Immunofluorescence images of WT and *Zbp1*^*−/−*^ primary MPVECs 48 h after co-treatment with LPS and rmCIRP (scale bar = 5 μm). Arrowheads indicate the PANoptosome. The bar chart displays the quantification of the percentage of cells with ASC^+^CASP8^+^RIPK3^+^ specks among the ASC speck^+^ cells. The data are presented as mean ± SD. ^***^P < 0.001. rmCIRP recombinant mouse cold-inducible RNA-binding protein, MPVEC mouse pulmonary vascular endothelial cell, GSDMD gasdermin D, CASP1 caspase-1, AIM2 absent in melanoma 2, CASP3 caspase-3, CASP8 caspase-8, p-MLKL phosphorylated mixed lineage kinase domain-like protein, MLKL mixed lineage kinase domain-like protein, RIPK3 receptor-interacting protein kinase 3, ASC apoptosis-associated speck-like protein containing a CARD

The inducibility of ZBP1 in WT MPVECs upon exposure to LPS and rmCIRP challenges was confirmed as shown in Fig. 5a. We assessed the role of ZBP1 in eCIRP-mediated PANoptosis by using ZBP1-deficient cells following the treatments of LPS and/or rmCIRP. The genetic ablation of ZBP1 significantly reduced cell death induced by LPS and rmCIRP (Fig. 5b).

Furthermore, we delineated how the deficiency of ZBP1 affects cell death pathways. Upon treatment with LPS and rmCIRP, there was a reduction in the cleavage of GSDMD, CASP1, AIM2, the P17 subunit of CASP3, and the P18 subunit of CASP8, along with decreased phosphorylation of MLKL in *Zbp1*^*−/−*^ MPVECs compared to WT cells (Fig. 5c). Previous studies have reported that ZBP1 regulates inflammasome activation in response to pathogens or thermal stress [20, 21], and inflammasomes can function as components of PANoptosomes that drive PANoptosis [20]. Therefore, we investigated the activation of inflammasomes following LPS and rmCIRP treatments in WT and *Zbp1*^*−/−*^ MPVECs. The formation of the PANoptosome complex, indicated by the colocalization of ASC specks with CASP8 and RIPK3, was significantly disrupted in the absence of ZBP1. WT cells showed pronounced colocalization, while *Zbp1*^*−/−*^ cells exhibited a notable decrease in PANoptosome complex formation (Fig. 5d). Collectively, these findings establish ZBP1 as a central regulator of inflammatory cell death.

### ZBP1 mediates mitochondrial damage and cell death, contributing to mortality in *sepsis*-induced ALI in vivo

**Fig. 6:**
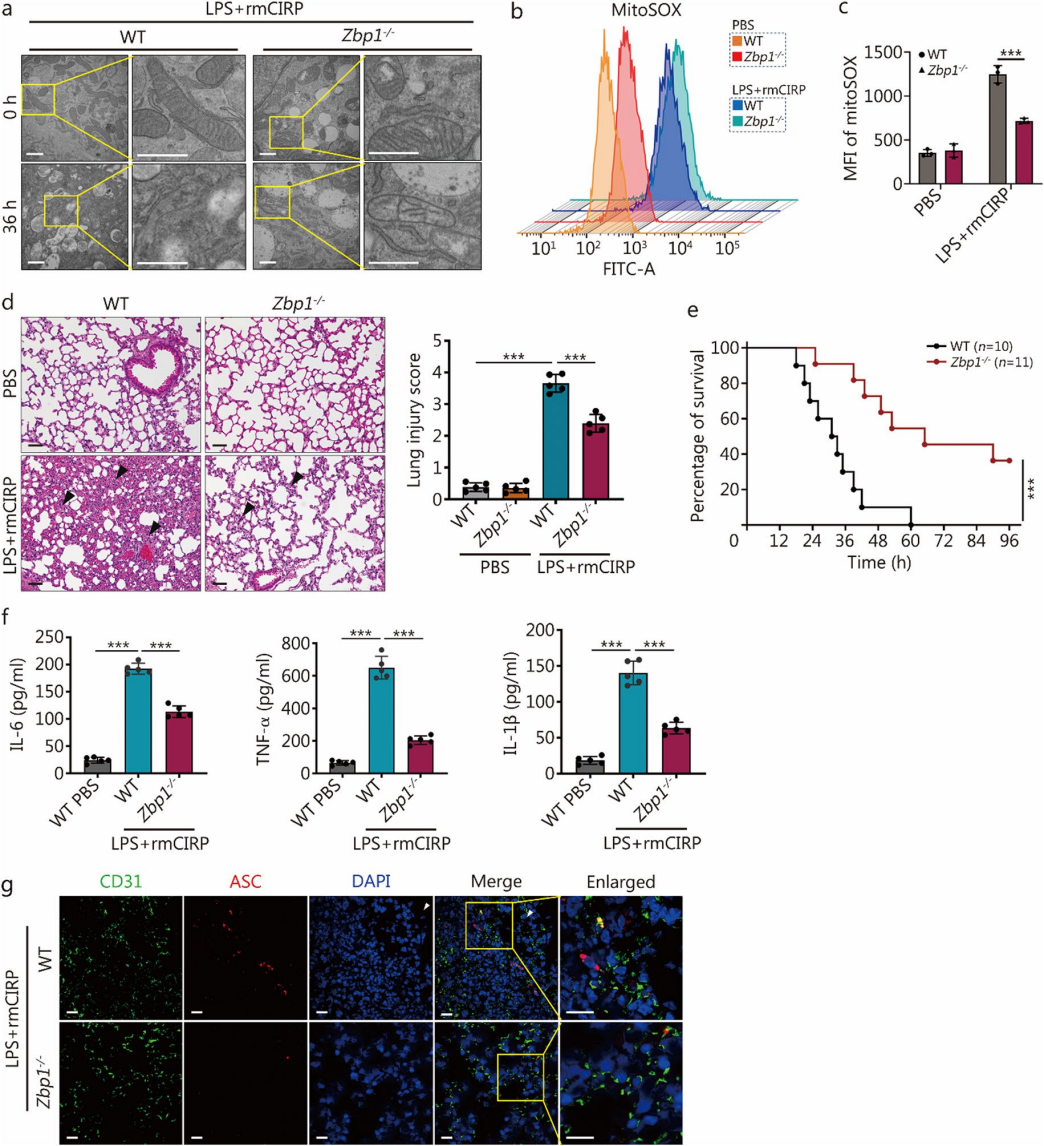
Z-DNA binding protein 1 (ZBP1) mediates mitochondrial damage and cell death, contributing to mortality in sepsis-induced acute lung injury (ALI) in vivo*.* **a-g** Intratracheal instillation of LPS at 3 mg/kg, intravenous administration of rmCIRP at 5 mg/kg, or PBS as a control were used. TEM revealed mitochondrial structure in primary MPVECs from WT and *Zbp1*^*−/−*^ mice at 0 h or 36 h post-treatment with lipopolysaccharide (LPS) and recombinant mouse CIRP (rmCIRP) (**a**, scale bar = 1 μm**)**. Mitochondrial ROS levels were measured using MitoSOX, a fluorogenic dye specifically targeted to mitochondria to detect superoxide production. The measurements were conducted using flow cytometry (**b**). Analyses of Annexin V/PI staining with flow cytometry detected apoptosis (**c**). Histological assessment of lung tissue from WT and *Zbp1*^*−/−*^ mice 36 h post-treatment with PBS or LPS + rmCIRP included H&E staining, arrows indicate lung injury characterized by alveolar septal thickening and inflammatory cell infiltration and a corresponding histological score (**d**, scale bar = 50 μm). Survival rates of male WT (*n* = 10) and *Zbp1*^*−/−*^ (*n* = 11) mice ages 6 – 8 weeks at 96 h post intravenous CIRP injection and intratracheal LPS instillation (**e**). Levels of the inflammatory cytokine IL-6, TNF-α, and IL-1β in BALF were measured 36 h after intravenous CIRP injection and intratracheal LPS instillation (**f**). Immunofluorescence quantified ASC-positive cells within CD31-marked pulmonary vascular endothelial cells in mouse lung tissue. White arrowheads indicate ASC specks formation within CD31^+^ cells (**g**, scale bar = 20 μm). Data are presented as mean ± SD. ^***^P < 0.001. TEM transmission electron microscopy, ROS reactive oxygen species, MitoSOX mitochondrial superoxide indicator (fluorogenic dye), PI propidium iodide, TNF-α tumor necrosis factor-α, IL-6 interleukin 6, IL-1β interleukin 1β, BALF bronchoalveolar lavage fluid, MPVEC mouse pulmonary vascular endothelial cell, MFI mean fluorescence intensity

A recent study indicates that mitochondrial damage is critical for cell death [33]. To elucidate the role of ZBP1 in the pathogenesis of sepsis-induced ALI in vivo, we administered rmCIRP intravenously alongside a non-lethal dose of LPS via intratracheal instillation in mice. In WT mice, the co-treatment with LPS and rmCIRP resulted in significant mitochondrial structural abnormalities, mainly characterized by cristae disruption, swelling, and vacuolation. Additionally, there was an increase in mitochondrial reactive oxygen species (ROS) in MPVECs, indicating mitochondrial impairment. This dysfunction was significantly reduced in *Zbp1*^*−/−*^ mice, highlighting the pivotal role of ZBP1 in the progression of mitochondrial-mediated cell death during sepsis (Fig. 6a–c).

Histological examination of lung tissue revealed severe lung injury after co-treatment with LPS and rmCIRP in WT mice, while *Zbp1*^*−/−*^ mice exhibited preserved pulmonary architecture and lower scores of lung injury (Fig. 6d). Survival analysis in the sepsis-induced ALI demonstrated that *Zbp1*^*−/−*^ mice had a markedly higher survival rate compared to WT mice (Fig. 6e).

Figure 6f illustrates that *Zbp1*^*−/−*^ suppressed the increase of IL-6, tumor necrosis factor-α (TNF-α), and IL-1β concentration in BALF in the WT mice upon septic challenge, implicating the role of ZBP1 in exacerbating the inflammatory cascade associated with ALI. Immunofluorescence staining revealed the formation of ASC specks colocalized with the EC marker CD31 in WT mice, which were notably absent in *Zbp1*^*−/−*^ mice (Fig. 6g), suggesting a reduction in inflammasomes and MPVECs death responses due to the absence of ZBP1. The colocalization of ASC with CD31 indicates that inflammasome activation occurs specifically in ECs, thereby contributing to both the inflammatory process and cell death pathways within vascular endothelium during sepsis-induced ALI.

Taken together, these results demonstrate that ZBP1-mediated PANoptosis contributes to pathology and mortality associated with sepsis-induced ALI, highlighting the significance of ZBP1 in the pathogenesis of this disease.

### ZBP1-activated RIPK3 is required for MPVEC PANoptosome

**Fig. 7:**
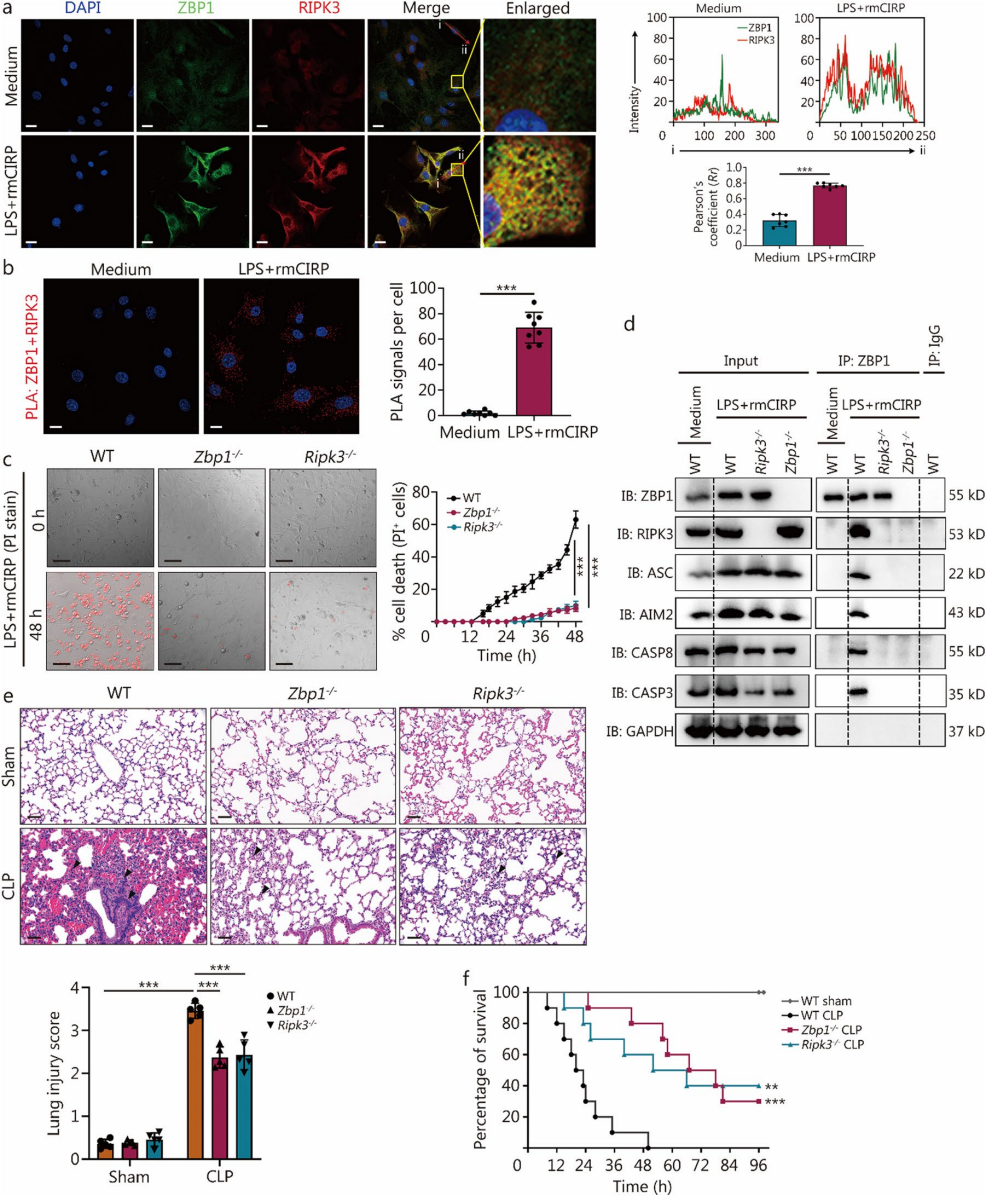
Z-DNA binding protein 1 (ZBP1)-activated receptor-interacting protein kinase 3 (RIPK3) is required for pulmonary MPVECs PANoptosome. **a** Immunofluorescence images of wild type (WT) primary MPVECs at 36 h post co-treatment with lipopolysaccharide (LPS) and recombinant mouse CIRP (rmCIRP) show significant co-localization of ZBP1 (green) and RIPK3 (red) (scale bar = 20 μm). The arrows from “i” to “ii” highlight the region analyzed for colocalization analysis. The images on the right show the analysis of ZBP1 and CIRP co-localization in immunofluorescence and Pearson’s correlation coefficient calculations. **b** Proximity ligation assay (PLA) revealed physical interactions between ZBP1 and RIPK3 as red spots in WT primary MPVECs 36 h after co-treatment with LPS and rmCIRP (scale bar = 20 μm). **c** Representative images of cell death and real-time analysis of cell death in WT, *Zbp1*^*−/−*^, and *Ripk3*^*−/−*^ primary MPVECs co-treated with LPS and rmCIRP (scale bar = 100 μm). **d** Immunoblot analysis of ZBP1, RIPK3, ASC, AIM2, CASP8, and CASP3 following immunoprecipitation (IP) with anti-ZBP1 or control IgG antibodies in WT, *Zbp1*^*−/−*^, and *Ripk3*^*−/−*^ primary MPVECs after 36 h of co-treatment with LPS and rmCIRP. **e** Hematoxylin and eosin (H&E)-stained lung tissues from WT, *Zbp1*^*−/−*^, and *Ripk3*^*−/−*^ mice at 36 h post-CLP (scale bar = 50 μm), arrows indicate lung injury characterized by alveolar septal thickening and inflammatory cell infiltration. A corresponding quantification of histology scores is provided below. **f** Survival analysis of male WT, *Zbp1*^*−/−*^, and *Ripk3*^*−/−*^ mice aged 6–8 weeks following CLP (n = 10) revealed significantly different survival rates in *Zbp1*^*−/−*^ and *Ripk3*^*−/−*^ mice compared to WT mice. Data are presented as mean ± SD. ^**^P < 0.01, ^***^P < 0.001. MPVEC mouse pulmonary vascular endothelial cell, ASC apoptosis-associated speck-like protein containing a CARD, AIM2 absent in melanoma 2, CASP8 caspase-8, CASP3 caspase-3

ZBP1 has been reported as an upstream effector in mediating PANoptosome formation, which executes PANoptosis [21]. In the present study, we showed that LPS and rmCIRP induced a notable colocalization of ZBP1 with RIPK3 in MPVECs (Fig. 7a, b), suggesting the initiation of ZBP1 PANoptosome complex assembly. Figure 7c further illustrates that *Zbp1*^*−/−*^ prevented the MPVECs’ death in response to LPS and rmCIRP, while the deletion of *RIPK3* also led to a reduction in cell death.

IP assays in primary MPVECs from WT, *Zbp1*^*−/−*^, and *Ripk3*^*−/−*^ genotypes revealed the formation of a multiprotein PANoptosome complex, comprising RIPK3, ASC, AIM2, CASP8, and CASP3 in the presence of ZBP1. The absence of either *Zbp1* or *Ripk3* disrupted this complex formation (Fig. 7d), indicating that the inflammasome components are integrated within the multiprotein PANoptosome complex and response to LPS and rmCIRP stimulation to drive inflammatory cell death.

Utilizing the in vivo CLP model, we found that the deficiency of *Zbp1* and *Ripk3* attenuated the severity of sepsis-induced ALI (Fig. 7e). Survival analysis conducted post-CLP showed that the mice with deficient *Zbp1* and *Ripk3* exhibited elevated survival rates compared to WT mice (Fig. 7f).

Collectively, ZBP1-mediated signaling induces the interaction between RIPK3 and CASP8 with ASC to form the ZBP1 PANoptosome, which triggers PANoptosis in response to LPS and rmCIRP.

### TRIM32 is a ZBP1 E3 ubiquitin (Ub) ligase destabilizing ZBP1

**Fig. 8:**
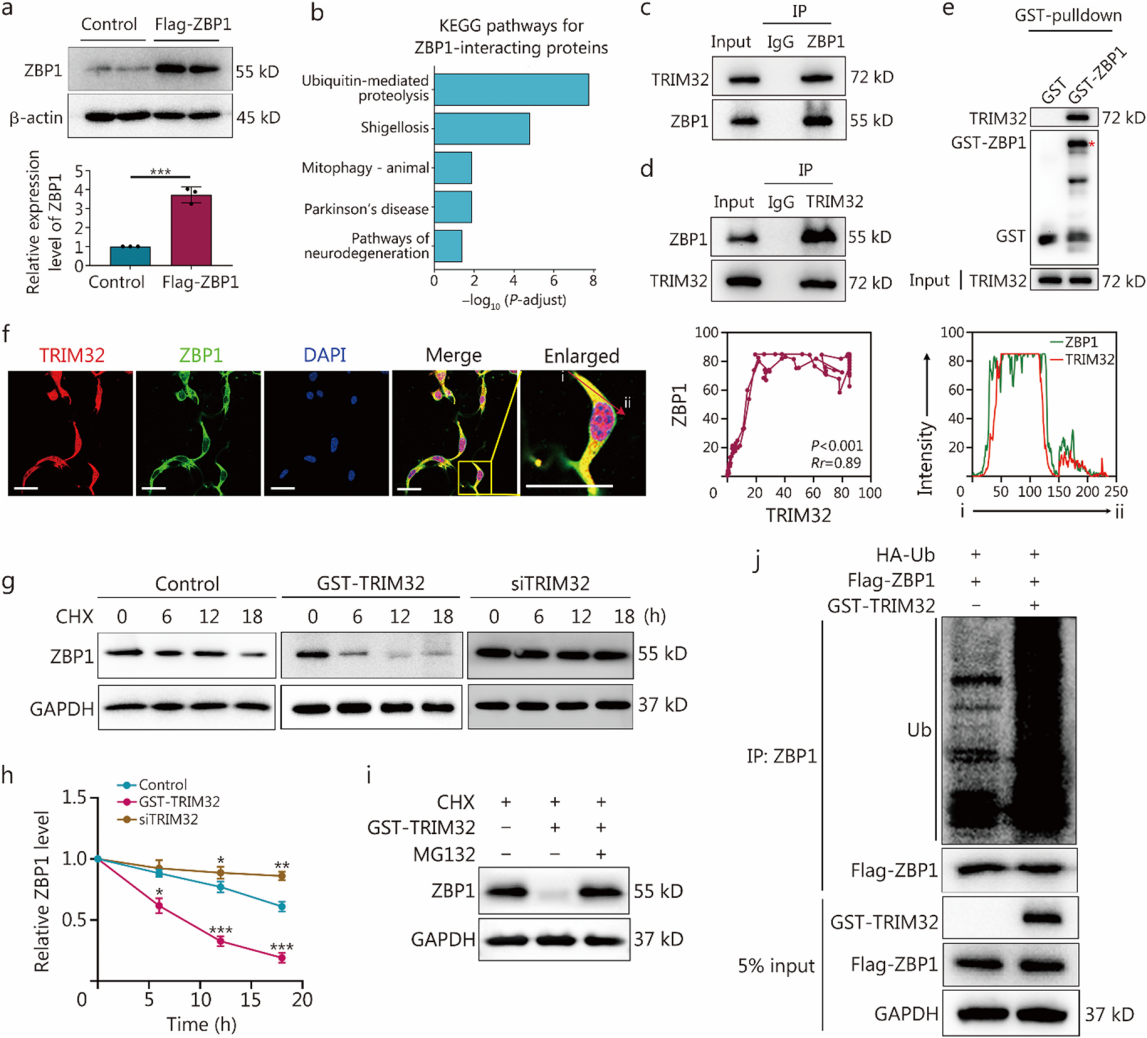
Tripartite motif containing 32 (TRIM32) is a Z-DNA binding protein 1 (ZBP1) E3 ubiquitin (Ub) ligase destabilizing ZBP1. **a** Western blotting analysis showing the expression of Flag-tagged ZBP1 in endothelial cells transfected with a Flag-ZBP1 plasmid, indicating a significant overexpression of ZBP1 compared to the control group. Quantification of the ZBP1 expression level is presented in the bar graph below with a significant increase in ZBP1 expression. **b** Kyoto Encyclopedia of Genes and Genomes (KEGG) pathway analysis of ZBP1-interacting proteins identified through ZBP1 immunoprecipitation followed by mass spectrometry from endothelial cells expressing ZBP1. The graph displays the enriched pathways, with Ub-mediated proteolysis being the most significant. **c** Co-IP assay from endothelial cell lysates using an anti-ZBP1 antibody, followed by immunoblotting for TRIM32 and ZBP1. The input and IP samples demonstrate the interaction between ZBP1 and TRIM32. **d** Lung vascular endothelial cells were transfected with a TRIM32 plasmid, and cell lysates were immunoprecipitated with an anti-TRIM32 antibody, followed by ZBP1 immunoblotting. **e** A GST-pulldown assay confirming a direct interaction between GST-tagged TRIM32 and ZBP1, as indicated by the presence of ZBP1 in the pulldown complex. **f** Immunofluorescence staining of TRIM32 (red) and ZBP1 (green) in endothelial cells, with nuclei counterstained with DAPI (blue). The merged image and the enlarged panel from the highlighted box show the colocalization of TRIM32 and ZBP1 (scale bar = 20 μm). Line graphs on the right display fluorescence intensity profiles for ZBP1 and TRIM32, corroborating their colocalization within the cells. The arrow from “i” to “ii” highlights the region analyzed for colocalization analysis. **g** Stability of ZBP1 protein was assessed over time by Western blotting in control cells, cells expressing GST-TRIM32 and *TRIM32* knockdown, treated with cycloheximide (CHX) to inhibit new protein synthesis. **h** The plot below the blots quantifies ZBP1 levels, revealing the effects of GST-TRIM32 overexpression and siTRIM32 knockdown on ZBP1 stability. **i** Western blotting analysis of ZBP1 levels in the presence of CHX with and without MG132 treatment in the GST-TRIM32 overexpression group. The symbols *, **, and *** indicate statistically significant differences compared to the control group. ^*^P < 0.05, ^**^P < 0.01, ^***^P < 0.001. **j** Ubiquitination assay depicting the modification of ZBP1 in the presence of HA-tagged Ub and GST-TRIM32. The smear of high molecular weight bands above ZBP1 indicates polyubiquitination. The assay was conducted using an anti-Flag antibody for immunoprecipitation, and ubiquitination levels were assessed by anti-HA Western blotting. Expression levels of TRIM32 and ZBP1 were verified by analyzing 5% of the input from cell lysates. The data are presented as the mean ± SD. MG132 a proteasome inhibitor, Co-IP co-immunoprecipitation

To identify a regulatory molecule that influences the ZBP1 functions, we employed proteomic screening to isolate a cohort of putative ZBP1 interactors. Initially, MPVECs were transfected with a Flag-ZBP1 plasmid to establish an overexpressing ZBP1 cell line (Fig. 8a). Immunoprecipitates of ZBP1 from the lysates of these cells were then analyzed via mass spectrometry to identify interacting partners (Additional file 2). Kyoto Encyclopedia of Genes and Genomes (KEGG) pathway analysis of these proteins indicated a significant enrichment in pathways such as ubiquitin-mediated proteolysis, prompting us to focus on ubiquitination-related biological processes (Fig. 8b).

To validate the results of immunoprecipitation followed by mass spectrometry, co-IP assays were conducted in a homologous system, with particular emphasis on the E3 Ub ligase TRIM32. Direct interaction between TRIM32 and ZBP1 was confirmed through these assays (Fig. 8c), with reciprocal IP further substantiating the specificity of this interaction (Fig. 8d). Furthermore, a glutathione S-transferase (GST) affinity isolation assay determined that ZBP1 directly interacted with TRIM32, as evidenced by TRIM32 co-purifying with GST-ZBP1 (Fig. 8e), thereby establishing a physical association between ZBP1 and TRIM32.

Immunofluorescence studies demonstrated the colocalization of TRIM32 with ZBP1 in cells, supported by line scan analysis that confirmed their proximity, indicative of a functional interaction within a cellular milieu (Fig. 8f). Pharmacological inhibition of de novo protein synthesis with cycloheximide (CHX, 100 μmol/L) resulted in a time-dependent decrease in ZBP1 protein levels, which was more pronounced in cells overexpressing TRIM32 (Fig. 8g). In contrast, ZBP1 protein levels remained significantly more stable in cells with *TRIM32* knockdown (Fig. 8h). This suggests that TRIM32 expedites the ubiquitination-dependent degradation of ZBP1. Furthermore, in the presence of CHX, treatment with the proteasome inhibitor MG132 markedly ameliorated the decrease in ZBP1 levels caused by GST-TRIM32 overexpression, indicating the role of proteasome-mediated degradation in ZBP1 ubiquitination (Fig. 8i). Moreover, in vitro ubiquitination assays elucidated that GST-TRIM32 overexpression significantly amplified ZBP1 polyubiquitination, providing evidence of TRIM32’s E3 ligase activity targeting ZBP1 (Fig. 8j).

These results reinforce the function of TRIM32 as an E3 Ub ligase that facilitates the degradation of ZBP1, highlighting its significant role as a post-translational modulator of this protein.

### eCIRP blocks TRIM32-mediated polyubiquitination and proteasomal degradation of ZBP1

**Fig. 9:**
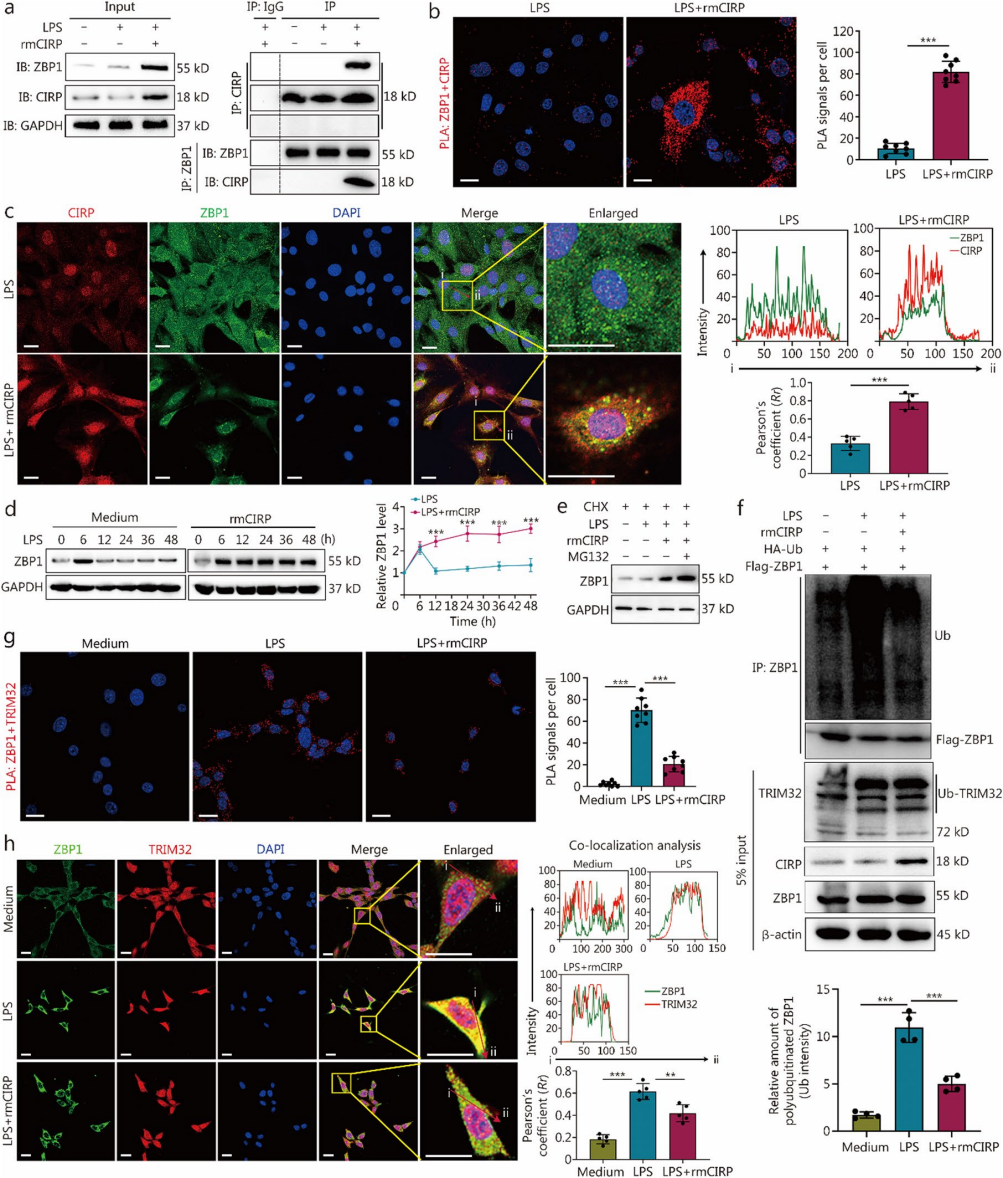
eCIRP blocks TRIM32-mediated polyubiquitination and proteasomal degradation of ZBP1. **a** Co-IP assays demonstrate the interaction between ZBP1 and CIRP in cells treated with LPS alone or LPS with rmCIRP for 12 h. The presence of CIRP and ZBP1 in the precipitated complexes is confirmed by Western blotting. **b** Proximity ligation assay (PLA) reveals physical associations between ZBP1 and CIRP as red spots in WT primary MPVECs after 12 h of co-treatment with LPS alone or LPS and rmCIRP (scale bar = 20 μm). The bar chart shows the quantitative analysis of PLA signals from panel B shows a significant increase in ZBP1-CIRP association when co-treated with LPS and rmCIRP compared to LPS alone. **c** Immunofluorescence images display the localization of CIRP (red) and ZBP1 (green) in cells treated with LPS, with and without rmCIRP treatment. The merged and enlarged images highlight the colocalization of CIRP and ZBP1, which is enhanced with rmCIRP treatment (scale bar = 20 μm). The right images show the fluorescence intensity profiles for CIRP and ZBP1 along the lines marked “i” and “ii” that support the colocalization observation. The bar graph quantifies the colocalization coefficients, showing a significant increase with LPS and rmCIRP treatment. **d** Western blotting analysis monitors the stability of ZBP1 protein over time in the presence of LPS and LPS + rmCIRP, indicating that rmCIRP treatment enhances ZBP1 stability compared to LPS treatment alone. A line graph quantifying the relative ZBP1 levels suggests that rmCIRP preserves ZBP1 stability against LPS-induced degradation. The symbol *** indicates statistically significant differences compared to the control group. ^***^P < 0.001. **e** Western blotting analysis to monitor the stability of ZBP1 protein following CHX inhibition of protein synthesis in the presence of LPS, LPS + rmCIRP, and MG132. **f** Ubiquitination assays for ZBP1, conducted with and without rmCIRP treatment in the presence of HA-tagged ubiquitin (HA-Ub), reveal a pattern of polyubiquitination. The extent of ZBP1 ubiquitination is determined by immunoblotting using antibodies specific for HA-tagged polyubiquitin conjugates. The expression levels of TRIM32, CIRP, and ZBP1 are confirmed by analyzing 5% of the input from cell lysates. The bar chart below displays the quantification of polyubiquitinated ZBP1 levels, normalized to the control. **g** PLA detects physical associations between ZBP1 and TRIM32 as red spots in MPVECs after 12 h of co-treatment with LPS and rmCIRP or LPS alone (scale bar = 20 μm). The right image shows the quantitative analysis of PLA signals revealing a significant decrease in ZBP1-TRIM32 association with LPS and rmCIRP co-treatment compared to LPS alone. **h** Immunofluorescence images show the localization of TRIM32 (red) and ZBP1 (green) in cells treated with LPS, with and without rmCIRP treatment for 12 h. The merged and enlarged images illustrate the colocalization of TRIM32 and ZBP1 (scale bar = 20 μm). Fluorescence intensity profiles for TRIM32 and ZBP1 along the lines marked “i” and “ii” in the right mages support the colocalization data. The bar graph quantifies the colocalization coefficients, showing a significant reduction with LPS and rmCIRP treatment compared to LPS treatment alone. The data are presented as the mean ± SD. ^**^P < 0.01, ^***^P < 0.001. eCIRP extracellular cold-inducible RNA-binding protein, TRIM32 tripartite motif-containing 32, ZBP1 Z-DNA binding protein 1, LPS lipopolysaccharide, rmCIRP recombinant mouse cold-inducible RNA-binding protein, PLA proximity ligation assay, WT wild type, MPVEC mouse pulmonary vascular endothelial cell, CHX cycloheximide, MG132 proteasome inhibitor, HA-tag hemagglutinin-tag, HA-Ub hemagglutinin-tagged ubiquitin, Co-IP co-immunoprecipitation

To address how rmCIRP promotes ZBP1 signaling, we aimed to determine whether CIRP directly interacts with ZBP1. Co-IP assays revealed the formation of CIRP and ZBP1 protein complexes in cell precipitates stimulated by co-treatments with LPS and rmCIRP (Fig. 9a). This interaction was further confirmed by PLA and confocal immunofluorescence microscopy. We observed a significant increase in the colocalization of ZBP1 with eCIRP in the cytoplasm of MLVECs treated with rmCIRP and LPS, as compared to the cells treated with LPS alone (Fig. 9b, c).

Protein stability assays, following CHX treatment and measured by Western blotting, showed an initial increase in ZBP1 levels at 6 h after LPS stimulation, which was subsequently followed by a decrease over time. The presence of eCIRP, however, attenuated the decline of ZBP1, indicating that eCIRP confers protection against LPS-induced ZBP1 degradation (Fig. 9d). Additionally, the presence of the proteasome inhibitor MG132 significantly increased ZBP1 levels following CHX-mediated inhibition of protein synthesis, suggesting that ZBP1 is subject to proteasomal degradation (Fig. 9e). Upon LPS stimulation, we noted the ubiquitination of TRIM32, which was absent under normal conditions. IP results demonstrated an increase in ZBP1 polyubiquitination levels upon LPS stimulation, while the addition of eCIRP significantly reduced the LPS-induced polyubiquitination of ZBP1 by Ub-TRIM32 (Fig. 9f), underscoring eCIRP’s inhibitory effect on ZBP1 ubiquitination during sepsis.

Importantly, PLA and confocal immunofluorescence microscopy showed a significant reduction in the binding of ZBP1 to TRIM32 in the presence of eCIRP under LPS-induced conditions (Fig. 9g, h). These data indicate that eCIRP shields ZBP1 from TRIM32-mediated ubiquitination and subsequent proteasomal degradation, thus promoting ZBP1-mediated cell death.

## Discussion

**Fig. 10:**
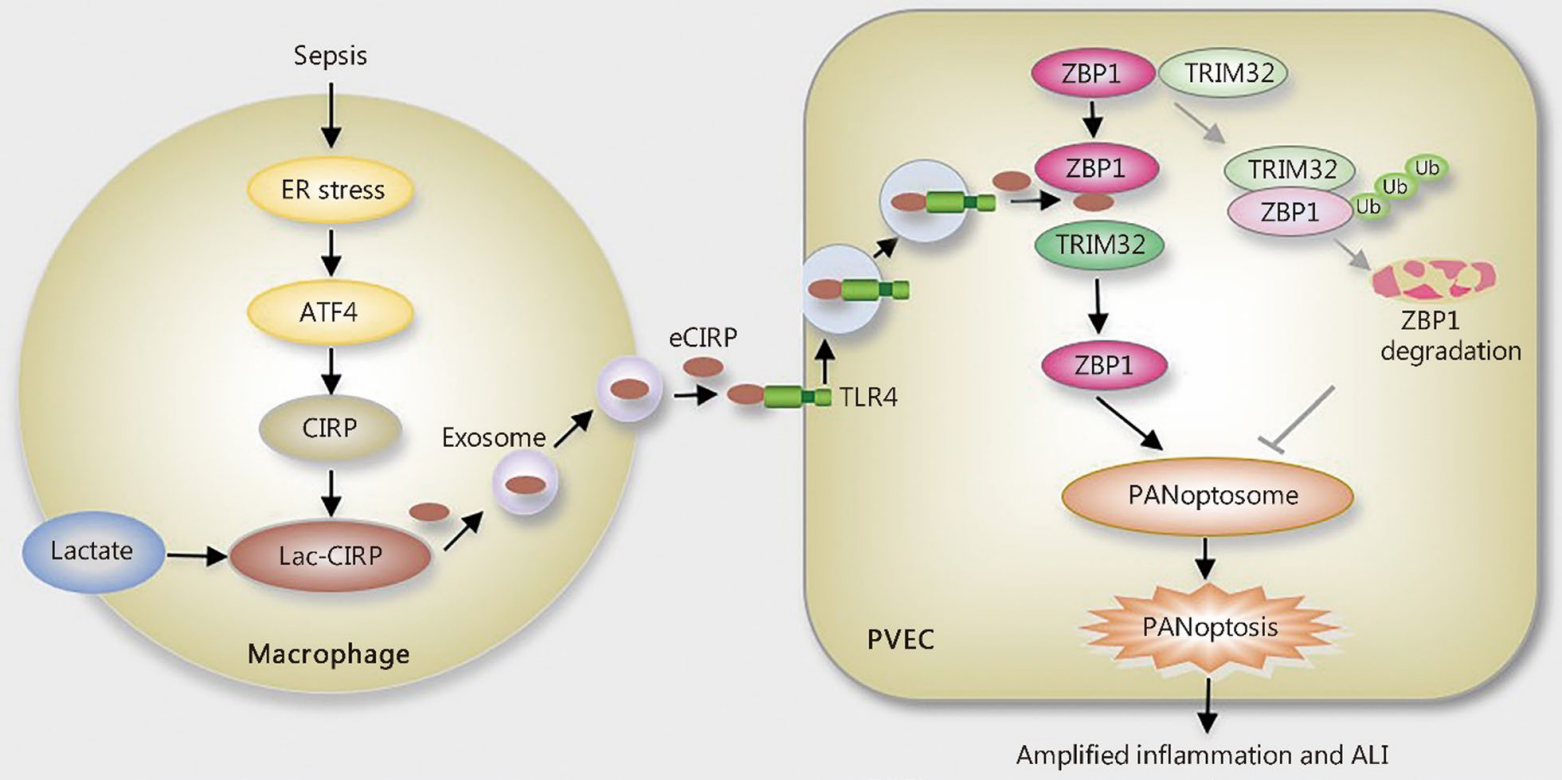
The current study demonstrates that ER stress, induced by sepsis or LPS stimulation in macrophages, triggers ATF4 activation, which subsequently enhances CIRP expression. The lactylation of CIRP (Lac-CIRP), a direct consequence of the septic upsurge in intracellular lactate, facilitates its migration from the nucleus to the cytoplasm and its subsequent release. The eCIRP is then internalized by PVECs through a TLR4-mediated endocytosis pathway and binds to ZBP1. This binding obstructs ZBP1’s interaction with TRIM32 and prevents TRIM32-mediated ZBP1 proteasomal degradation. This interference preserves ZBP1 stability, which in turn amplifies ZBP1-dependent PVEC PANoptosis and exacerbates ALI. ER endoplasmic reticulum, LPS lipopolysaccharide, ATF4 activating transcription factor 4, CIRP cold-inducible RNA-binding protein, eCIRP extracellular cold-inducible RNA-binding protein, PVEC pulmonary vascular endothelial cell, TLR4 Toll-like receptor 4, ZBP1 Z-DNA binding protein 1, TRIM32 tripartite motif-containing 32, PANoptosis a form of programmed cell death that combines elements of pyroptosis apoptosis and necroptosis, ALI acute lung injury

Based on the presented findings, we propose that ER stress, induced by sepsis or LPS stimulation in macrophages, triggers ATF4 activation, which subsequently enhances the expression of CIRP. The lactylation of CIRP, a direct consequence of the septic upsurge in intracellular lactate, facilitates its migration from the nucleus to the cytoplasm and subsequent release into the extracellular space. The eCIRP is internalized by PVECs through a TLR4-mediated endocytosis pathway and competitively binds to ZBP1, thereby obstructing its interaction with TRIM32 and preventing TRIM32-mediated proteasomal degradation of ZBP1. This interference preserves ZBP1 stability, which in turn amplifies ZBP1-RIPK3-dependent PVEC PANoptosis and exacerbates ALI (Fig. 10).

A previous study reported that higher plasma eCIRP levels are positively correlated with mortality and the severity of multiple organ dysfunction in patients with sepsis [8]. Our study found that eCIRP was significantly increased in the exosomes of plasma from CLP mice and positively correlated with lung injury. Recent research has shown that CIRP inhibitor C23 can reduce inflammatory response by targeting eCIRP, thereby significantly ameliorating organ tissue damage caused by sepsis and ischemia/reperfusion [34, 35]. Studies have also demonstrated that eCIRP can independently promote the progression of inflammation [7–9]. In cells under hypoxic conditions, CIRP translocates from its normal nuclear localization to the cytoplasm [14] before being released into the extracellular space [8]. eCIRP is capable of engaging various cell types to elicit cellular inflammatory responses, including inducing macrophages to release pro-inflammatory cytokines and vascular endothelial dysfunction [12, 13]. These findings support eCIRP as an important determinant in the regulation of inflammation under critically ill conditions. However, the cellular origin of eCIRP and its role in mediating ALI following sepsis remained unclear.

The active release of eCIRP is mainly mediated by lysosomes and exosomes, which are secreted by exocytosis [36]. Stressors such as oxidative stress, ER stress, and heat shock can trigger the migration of CIRP from the nucleus to the cytoplasm through a methylation-dependent mechanism [12]. Significantly, our findings reveal that lactate can induce the lactylation of CIRP, thereby enhancing its translocation from the nucleus to the cytoplasm and subsequent release via exosomes during sepsis. The process mediated by lactate involves histone lysine modification known as Klac [37], with existing research demonstrating that lactylation can regulate the cytoplasmic localization of HMGB1 [27]. Oxidative stress prompts CIRP’s migration to the cytoplasm without altering its expression levels [14]. However, previous studies have not fully elucidated the molecular mechanism underlying CIRP upregulation. It has been reported that excessive ER stress in the liver induces the activation of ATF4, thereby promoting the expression of CIRP [38]. In this study, we found that in sepsis, ER stress activates ATF4, which in turn upregulates CIRP expression. Notably, our data suggest that ATF4 may directly bind to the CIRP promoter and drive its expression.

Intercellular diffusion of DAMPs to adjacent cells can be sensed by intracellular receptors, with one primary pathway being through gap junctions [39]. Additionally, a previous study has also demonstrated that DAMPs can trigger pyroptosis via receptor-mediated endocytosis [40]. Our findings reveal that during sepsis, TLR4 serves as the primary receptor for eCIRP and plays a crucial role in mediating its internalization in ECs as shown in Fig. 3, although some other receptors, such as triggering receptor expressed on myeloid cells-1 and interleukin-6 receptor have been previously identified as eCIRP receptors and serve different roles under various physiological and pathological conditions [8, 9, 41]. Moreover, we have discovered that the dynamin-dependent endocytosis of CIRP activates a cascade of intracellular events leading to PANoptosis.

Our study demonstrates that eCIRP is transported into ECs via TLR4-mediated internalization. Research indicates that endocytosis of cell surface receptors constitutes a critical regulatory mechanism in signal transduction [42]. Following internalization, TLR4 translocates the endosomal network, where it triggers an alternative signaling pathway via the adaptors TRIF-related adaptor molecule and TRIF [29, 43]. Therefore, LPS-induced receptor endocytosis is crucial for the signaling function of TLR4 [28, 30].

We identify ZBP1-mediated signaling as an upstream regulator of RIPK3, which is essential for the formation of ZBP1 PANoptosome in MPVECs upon stimulation with LPS and eCIRP. This finding underscores the intricate regulatory network involving ZBP1 and RIPK3, wherein ZBP1 acts as a master regulator that initiates the assembly of PANoptosomes crucial for executing PANoptosis. These results are consistent with previous research delineating the role of ZBP1 in mediating cell death across diverse contexts, particularly during influenza A virus infection and heatstroke [21, 32]. Our findings reinforce these studies by emphasizing the significance of ZBP1 in the inflammatory response and cell death mechanisms associated with sepsis.

The interactions between proteins, both homotypic and heterotypic, serve as the foundational framework for the formation of PANoptosome [20, 44]. Prior research has identified ZBP1 and RIPK1 as key molecules initiating PANoptosome formation in response to specific stimuli [21], suggesting a complex network of interactions critical for this process. Necroptosis is regulated by RIP homotypic interaction motif interaction, while the heterotypic interaction between ASC and CASP8 integrates mechanisms of pyroptosis and apoptosis [45], forming the scaffold of PANoptosome. Furthermore, ASC specks representing inflammasome activation can colocalize with apoptotic and necrotic specks (CASP8 and RIPK3) in the same cell. Additional studies have shown that AIM2, pyrin, and ZBP1 are associated with ASC, CASP1, CASP8, RIPK3, RIPK1, and FADD to form a large multiprotein complex known as the PANoptosome that drives the unique inflammatory cell death termed PANoptosis [20, 46]. Our observations in sepsis-induced MPVEC death further support this model, highlighting the versatility of ZBP1 in engaging diverse cell death pathways.

Studies have highlighted the critical role of ZBP1 polyubiquitination in regulating cell death activated by the influenza virus [47, 48]. In this study, we identified the E3 Ub ligase TRIM32 as a pivotal regulator of ZBP1 proteasomal degradation. Intriguingly, in the context of sepsis, eCIRP impedes the interaction between TRIM32 and ZBP1, suppresses TRIM32-mediated polyubiquitination and proteasomal degradation of ZBP1, and thereby stabilizes its expression. This finding provides a novel perspective on the antagonistic interplay between TRIM32 and eCIRP in the post-translational regulation of ZBP1, offering a potential mechanism for the determination of cell fate towards PANoptosis during sepsis.

Nevertheless, we realize the limitations of this study. The specific competitive binding sites through which TRIM32 and eCIRP interact with ZBP1 warrant further exploration. Additionally, advanced techniques such as mass spectrometry and site-directed mutagenesis are essential for future investigations to pinpoint the precise ubiquitination sites on ZBP1. Furthermore, future studies involving conditional knockout models such as Cirpflox/floxLyzcre mice could greatly enhance our understanding of the macrophage-PVEC interactions during ALI and sepsis. Such investigations will enhance our comprehension of the intricate molecular mechanisms governing cell fate decisions in the context of sepsis.

## Conclusions

In summary, our study elucidates the mechanism by which excessive ER stress and ATF4 activation led to the upregulation of CIRP expression in macrophages. Furthermore, lactylation of CIRP and its subsequent release via exosomes meditates the regulatory role of the macrophage in PVEC death. Critically, during sepsis, internalized eCIRP in PVECs disrupts the interaction between ZBP1 and TRIM32, impeding proteasomal degradation of ZBP1 while stabilizing it. This stabilization enhances ZBP1-RIPK3-dependent PANoptosis, thereby exacerbating ALI. These insights offer novel perspectives and potential strategies for diagnosing and treating sepsis-induced ALI.

## Supplementary Information

**Additional file 1: Table S1** A list of antibodies used for Western blotting, IP, and immunofluorescence staining. **Table S2** For luciferase and mutant assay. **Table S3** Baseline characteristics of healthy controls and sepsis-induced ALI patients for cohort. **Table S4** Demographic and clinical characteristics of survivors and non-survivors. **Fig. S1** Expression distribution and single cell analysis. **Fig. S2** Evident endoplasmic reticulum stress in sepsis mice and cells. **Fig. S3** CIRP is released through the exosome pathway. **Fig. S4** LPS and rmCIRP induce PANoptosis in MPVECs.

**Additional file 2.**

## Data Availability

The datasets used or analyzed during the current study are available from the corresponding author upon reasonable request.

## Abbreviations

AIM2: Absent in melanoma 2
ALI: Acute lung injury
ASC: Apoptosis-associated speck-like protein containing a CARD
BALF: Bronchoalveolar lavage fluid
CASP1: Caspase-1
CASP3: Caspase-3
CASP8: Caspase-8
CIRP: Cold-inducible RNA-binding protein
CLP: Cecal ligation and puncture
DAMPs: Damage-associated molecular patterns
eCIRP: Extracellular cold-inducible RNA-binding protein
ER: Endoplasmic reticulum
FADD: Fas-associated protein with death domain
GST: Glutathione S-transferase
HMGB1: High mobility group box 1
i.p.: Intraperitoneally
Klac: Lysine lactylation
LPS: Lipopolysaccharide
MLVECs: Mouse lung vascular endothelial cells
MPVEC: Mouse pulmonary vascular endothelial cells
PI: Propidium iodide
PLA: Proximity ligation assay
PVECs: Pulmonary vascular endothelial cells
RIPK1: Receptor-interacting protein kinase 1
RIPK3: Receptor-interacting protein kinase 3
rmCIRP: Recombinant mouse cold-inducible RNA-binding protein
ROS: Reactive oxygen species
TLR4: Toll-like receptor 4
TNF-α: Tumor necrosis factor-α
TRIM32: Tripartite motif containing 32
TRIF: TIR-domain-containing adapter-inducing interferon-β
ZBP1: Z-DNA binding protein 1
