# Supplementary material for: Mechanism of lactic acidemia-promoted pulmonary endothelial cells death in sepsis: role for CIRP-ZBP1-PANoptosis pathway
Source: Mil Med Res. 2024 Oct 28;11:71. doi: 10.1186/s40779-024-00574-z (PMC11514876; doi:10.1186/s40779-024-00574-z)
Supplement: Supplementary file 1 — Additional file 1: Table S1 A list of antibodies used for Western blotting, IP, and immunofluorescence staining. Table S2 For luciferase and mutant assay. Table S3 Baseline characteristics of healthy controls and sepsis-induced ALI patients for cohort. Table S4 Demographic and clinical characteristics of survivors and non-survivors. Fig. S1 Expression distribution and single cell analysis. Fig. S2 Evident endoplasmic reticulum stress in sepsis mice and cells. Fig. S3 CIRP is released through the exosome pathway. Fig. S4 LPS and rmCIRP induce PANoptosis in MPVECs. [file 40779_2024_574_MOESM1_ESM.pdf]

Table S1 A list of antibodies used for Western blotting, IP, and immunofluorescence staining

Name of antibody	Catalog number	Company	Molecular weight (kD)	Dilution (Western blotting/IP/immunofluorescence)
CIRP	LS-C334809	LSBio	18	1:1000 (Western blotting); 1:50 (IP); 1:100 (immunofluorescence)
PERK	ab229912	Abcam	150	1:1000 (Western blotting)
p-PERK	PA5-40294	Invitrogen	150	1:1000 (Western blotting)
eIF2α	ab242148	Abcam	36	1:1000 (Western blotting)
p-eIF2α	ab32157	Abcam	38	1:1000 (Western blotting)
ATF4	#11815	CST	50	1:1000 (Western blotting)
CHOP	#2895	CST	27	1:1000 (Western blotting)
HSP70	ab2787	Abcam	70	1:1000 (Western blotting)
Histone H3	ab1791	Abcam	15	1:1000 (Western blotting)
Pan-lactate	SAB5701141	Sigma	35－100	1:1000 (Western blotting)
Klac	PTM1401RM	PTM BIO	15	1:1000 (Western blotting); 1:50 (IP); 1:100 (immunofluorescence)
CD31	#3528	CST	130	1:800 (immunofluorescence)
CD63	ab134045	Abcam	26	1:1000 (Western blotting)
CD81	ab109201	Abcam	26	1:1000 (Western blotting)
Calnexin	ab133615	Abcam	90	1:1000 (Western blotting)
CASP1	AG-20B-0042	Adipogen	45, 20	1:1000 (Western blotting); 1:100 (IP); 1:100 (immunofluorescence)
GSDMD	ab209845	Abcam	53, 34	1:1000 (Western blotting)
AIM2	# MA5-38442	Invitrogen	48	1:1000 (Western blotting); 1:100 (immunofluorescence)
F4/80	ab300421	Abcam	150	1:100 (immunofluorescence)
ASC	#67824	CST	22	1:1000 (Western blotting); 1:100 (immunofluorescence)
ZBP1	sc-271483	Santa Cruz	55	1:1000 (Western blotting); 1:50 (IP); 1:100 (immunofluorescence)
β-actin	#93473	CST	45	1:1000 (Western blotting)
GAPDH	5174	CST	37	1:1000 (Western blotting)
Tubulin	sc-32293	Santa Cruz	55	1:1000 (Western blotting)
TRIM32	NBP1-33737	Novus Biologicals	72	1:1000 (Western blotting); 1:50 (IP); 1:100 (immunofluorescence)

**Name of antibody**	**Catalog number**	**Company**	**Molecular weight (kD)**	**Dilution (Western blotting/IP/immunofluorescence)**
Ubiquitin	#43124	CST	-	1:1000 (Western blotting); 1:50 (IP)
MLKL	#70934	CST	54	1:1000 (Western blotting); 1:100 (immunofluorescence)
p-MLKL	#70934	CST	54	1:1000 (Western blotting)
Caspase-3	#70934	CST	35, 19, 17	1:1000 (Western blotting); 1:100 (immunofluorescence)
RIP3	#70934	CST	55	1:1000 (Western blotting); 1:100 (immunofluorescence)
p-RIP3	#70934	CST	55	1:1000 (Western blotting)
RIP1	ab300617	Abcam	75	1:1000 (Western blotting); 1:100 (immunofluorescence)
p-RIP1	ab316923	Abcam	75	1:1000 (Western blotting)
Cleaved caspase-8	#9496	CST	18	1:1000 (Western blotting); 1:100 (immunofluorescence)
Caspase-8	#4927	CST	55	1:1000 (Western blotting); 1:100 (immunofluorescence)

IP immunoprecipitation, *CIRP* cold-inducible RNA-binding protein, *PERK* protein kinase R (PKR)-like endoplasmic reticulum kinase, *p-PERK* phosphorylated protein kinase R (PKR)-like endoplasmic reticulum kinase, *eIF2α* eukaryotic initiation factor 2 alpha, *p-eIF2α* phosphorylated eukaryotic initiation factor 2 alpha, *ATF4* activating transcription factor 4, *CHOP* C/EBP homologous protein, *HSP70* heat shock protein 70, *CASP1* caspase-1, *GSDMD* gasdermin D, *AIM2* absent in melanoma 2, *ASC* apoptosis-associated speck-like protein containing a CARD, *ZBP1* Z-DNA binding protein 1, *TRIM32* tripartite motif-containing protein 32, *MLKL* mixed lineage kinase domain-like protein, *p-MLKL* phosphorylated mixed lineage kinase domain-like protein, *RIPK3* receptor-interacting protein kinase 3, *p-RIP3* phosphorylated receptor-interacting protein kinase 3, *RIP1* receptor-interacting protein kinase 1, *p-RIP1* phosphorylated receptor-interacting protein kinase 1

Table S2 For luciferase and mutant assay

Name	Sequence (5' – 3')	
pcDNA3.1ATF4 (1000 bp)	Forward	CGCGGATCCCGCAACATGACCGAAATGAGC
	Reverse	GGAATTCCAACTAGGGGACCCTTTTCTTCC
pGL4.1Cirp (1900 bp)	Forward	CGGGGTACCTCACAGAACCAGGGAACGATG
	Reverse	CTAGCTAGCCTCGGAGCCGGTGCAGCCAG
pGL4.1 Cirp-mutantA	Forward	CGGCAAGAAGCTTAGAATTATGCAG
	Reverse	CCTGCATAATTCTAAGCTTCTTGC
pGL4.1 Cirp-mutantB	Forward	CGTGAGTGGCATCGTGAGCAGC
	Reverse	CGGCTGCTCACGATGCCACTCAC
pGL4.1 Cirp-mutantC	Forward	GTCTTCCCGCAACGTACAGGGACC
	Reverse	AGGTCCCTGTACGTTGCGGGAAG
pGL4.1 Cirp-mutantABC	Forward	GCCTCCGATCGTTGTCAGAAG
	Reverse	GGTAGTCGGTCTTGCTATCCATG

Table S3 Baseline characteristics of healthy controls and sepsis-induced ALI patients for cohort

**Characteristic**	**Healthy controls[a] (*n* = 30)**	**Sepsis-induced ALI[a] (*n* = 60)**	***P*-value**
Age (years)	54 ± 3	55 ± 2	0.77
Gender [male, *n* (%)]	16 (53)	34 (57)	
Body mass index (kg/m^2)	22.3 ± 0.5	21.5 ± 0.4	0.215
Septic shock [*n* (%)]	-	32 (53)	
Source of infection [*n* (%)]			
Abdominal	-	44 (74)	
Urogenital	-	8 (13)	
Blood	-	5 (8)	
Wound	-	3 (5)	
Acute lung injury [*n* (%)]	-	60 (100)	
SOFA score, median (IQR)	-	8.0 (5.0 − 10.0)	
APACHE II score, median (IQR)	-	20.5 (16.0 − 25.8)	

[a]Confirmatory sample for ELISA. The values are presented as mean ± SD, median (IQR), or *n* (%). *APACHE II* Acute Physiology and Chronic Health Evaluation score II, SOFA Sequential Organ Failure Assessment, ALI acute lung injury

Table S4 Demographic and clinical characteristics of survivors and non-survivors

**Characteristic**	**Survivor (*n* = 22)**	**Non-survivor (*n* = 16)**	***P*-value**
Age (years)	55.4 ± 3.1	60.0 ± 2.9	0.31
Gender [male, *n* (%)]	10 (45)	10 (63)	
Body mass index (kg/m^2)	21.9 ± 0.6	20.4 ± 0.6	0.11
Septic shock [*n* (%)]	11 (50)	10 (63)	0.52
Source of infection [*n* (%)]			
Abdominal	16 (73)	12 (75)	
Urogenital	3 (13)	2 (13)	
Blood	2 (9)	1 (6)	
Wound	1 (5)	1 (6)	
Acute lung injury [*n* (%)]	22 (100)	16 (100)	
SOFA score, median (IQR)	7 (5 − 9)	11(9 − 13)	< 0.001
APACHE II score, median (IQR)	18 (12 − 25)	22 (19 − 26)	0.04

The values are presented as mean ± SD, median (IQR), or *n* (%). *APACHE II* Acute Physiology and Chronic Health Evaluation score II, *SOFA* Sequential Organ Failure Assessment

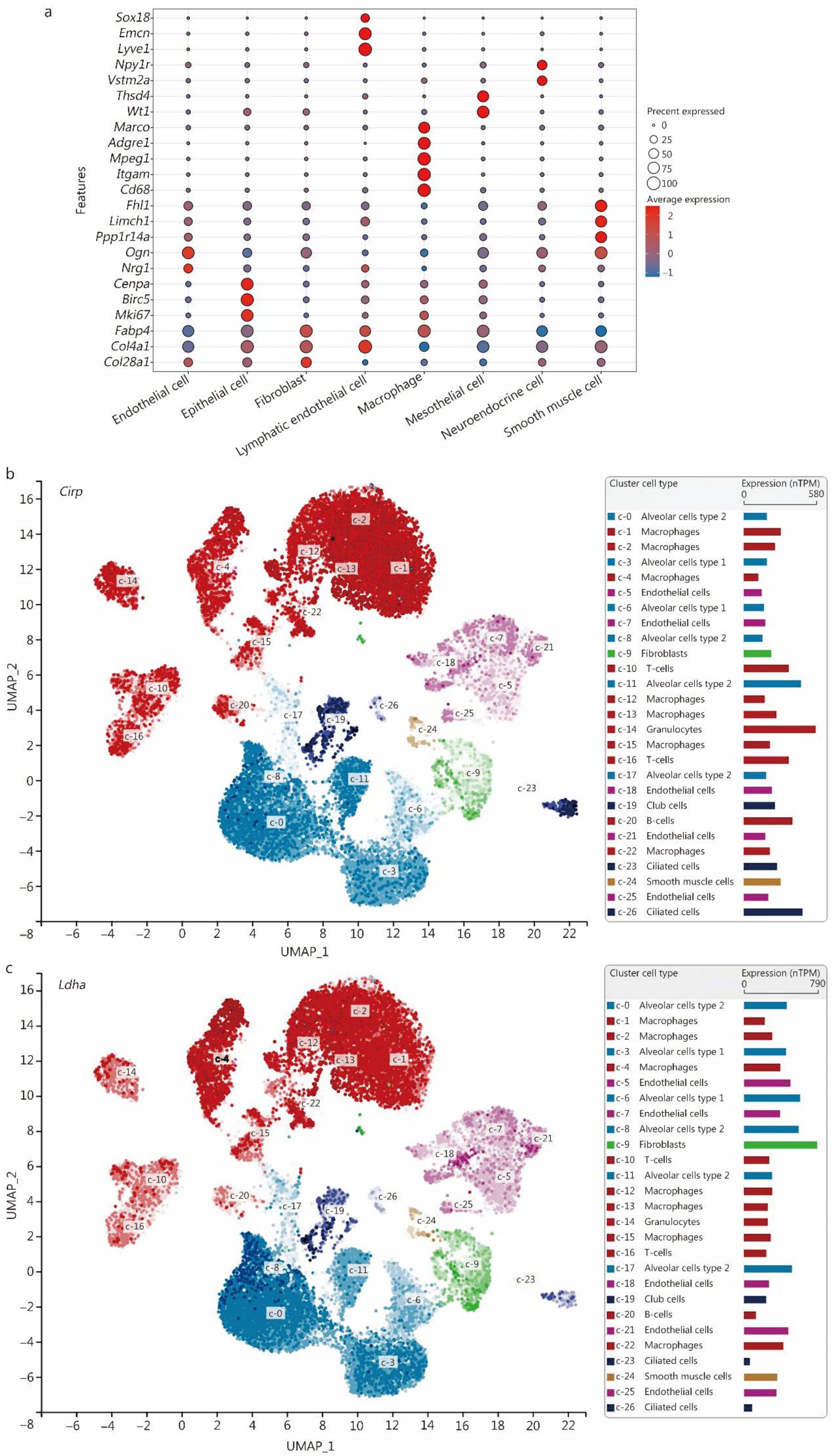

a
Sox18
Emcn
Lyve1
Npy1r
Vstm2a
Thsd4
Wt1
Marco
Adgre1
Mpeg1
Itgam
Cd68
Fhl1
Limch1
Ppp1r14a
Ogn
Nrg1
Cenpa
Birc5
Mki67
Fabp4
Col4a1
Col28a1
Features
Endothelial cell
Epithelial cell
Fibroblast
Lymphatic endothelial cell
Macrophage
Mesothelial cell
Neuroendocrine cell
Smooth muscle cell
Precent expressed
0
25
50
75
100
Average expression
2
1
0
−1
b
Cirp
UMAP_1
UMAP_2
Cluster cell type
Expression (nTPM)
0
580
c-0 Alveolar cells type 2
c-1 Macrophages
c-2 Macrophages
c-3 Alveolar cells type 1
c-4 Macrophages
c-5 Endothelial cells
c-6 Alveolar cells type 1
c-7 Endothelial cells
c-8 Alveolar cells type 2
c-9 Fibroblasts
c-10 T-cells
c-11 Alveolar cells type 2
c-12 Macrophages
c-13 Macrophages
c-14 Granulocytes
c-15 Macrophages
c-16 T-cells
c-17 Alveolar cells type 2
c-18 Endothelial cells
c-19 Club cells
c-20 B-cells
c-21 Endothelial cells
c-22 Macrophages
c-23 Ciliated cells
c-24 Smooth muscle cells
c-25 Endothelial cells
c-26 Ciliated cells
c
Ldha
UMAP_1
UMAP_2
Cluster cell type
Expression (nTPM)
0
790
c-0 Alveolar cells type 2
c-1 Macrophages
c-2 Macrophages
c-3 Alveolar cells type 1
c-4 Macrophages
c-5 Endothelial cells
c-6 Alveolar cells type 1
c-7 Endothelial cells
c-8 Alveolar cells type 2
c-9 Fibroblasts
c-10 T-cells
c-11 Alveolar cells type 2
c-12 Macrophages
c-13 Macrophages
c-14 Granulocytes
c-15 Macrophages
c-16 T-cells
c-17 Alveolar cells type 2
c-18 Endothelial cells
c-19 Club cells
c-20 B-cells
c-21 Endothelial cells
c-22 Macrophages
c-23 Ciliated cells
c-24 Smooth muscle cells
c-25 Endothelial cells
c-26 Ciliated cells

Fig. S1 Expression distribution and single cell analysis. **a** The single-cell RNA sequencing analysis demonstrated distinct cell clustering within lung tissue. Single-cell sequencing data from the Protein Atlas database indicates that the *Cirp* (**b**) and *Ldha* (**c**) genes are predominantly expressed in macrophages within lung tissue. Cirp cold-inducible RNA-binding protein, Ldha lactate dehydrogenase A

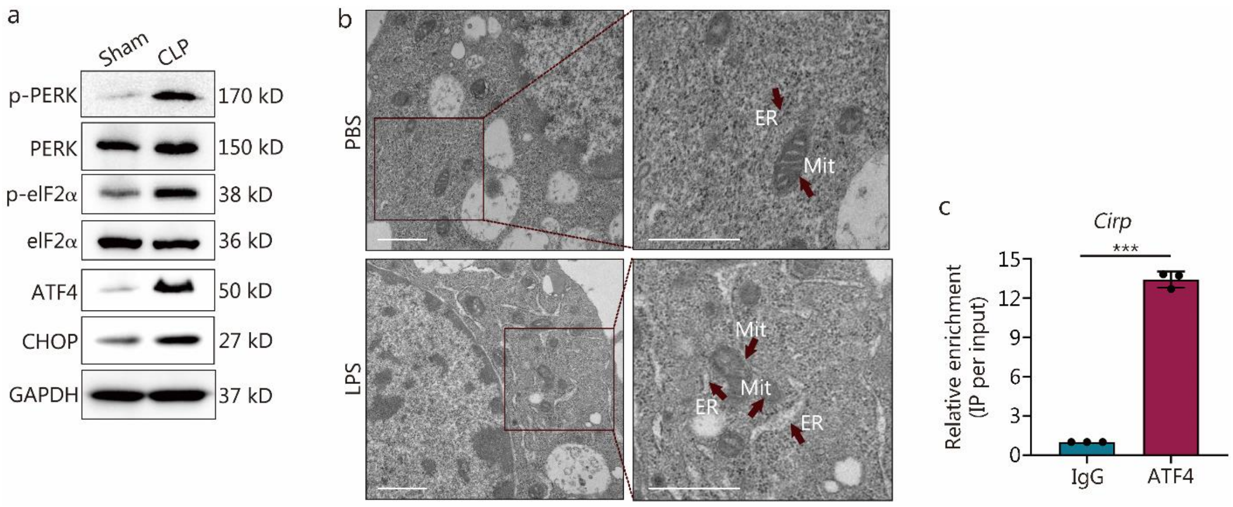

Fig. S2 Evident endoplasmic reticulum (ER) stress in sepsis mice and cells. **a** Immunoblotting was used to assess the expression of ER stress proteins p-PERK, PERK, p-eIF2α, eIF2α, ATF4, and CHOP in lung macrophages from CLP mice compared to the sham group. **b** Changes in mitochondrial morphology in macrophages after LPS stimulation were observed through electron microscopy (scale bar = 1 μm). The upper panel shows normal mitochondria and ER in the control group, indicated by arrows. The lower panel shows ER swelling and mitochondria vacuolation after LPS stimulation, indicated by arrows. **c** ChIP-qPCR analysis demonstrated that ATF4 binding to the *Cirp* promoter was significantly enriched compared to the IgG control. The results represent 3 independent experiments. The data are presented as the mean ± SD. $^{***}P < 0.001$. p-PERK phosphorylated protein kinase RNA-like endoplasmic reticulum kinase, PERK protein kinase RNA-like endoplasmic reticulum kinase, p-eIF2α phosphorylated eukaryotic initiation factor 2α, eIF2α eukaryotic initiation factor 2α, ATF4 activating transcription factor 4, CHOP C/EBP homologous protein, CLP cecal ligation and puncture, LPS lipopolysaccharide, ChIP-qPCR chromatin immunoprecipitation quantitative polymerase chain reaction, Mit mitochondria

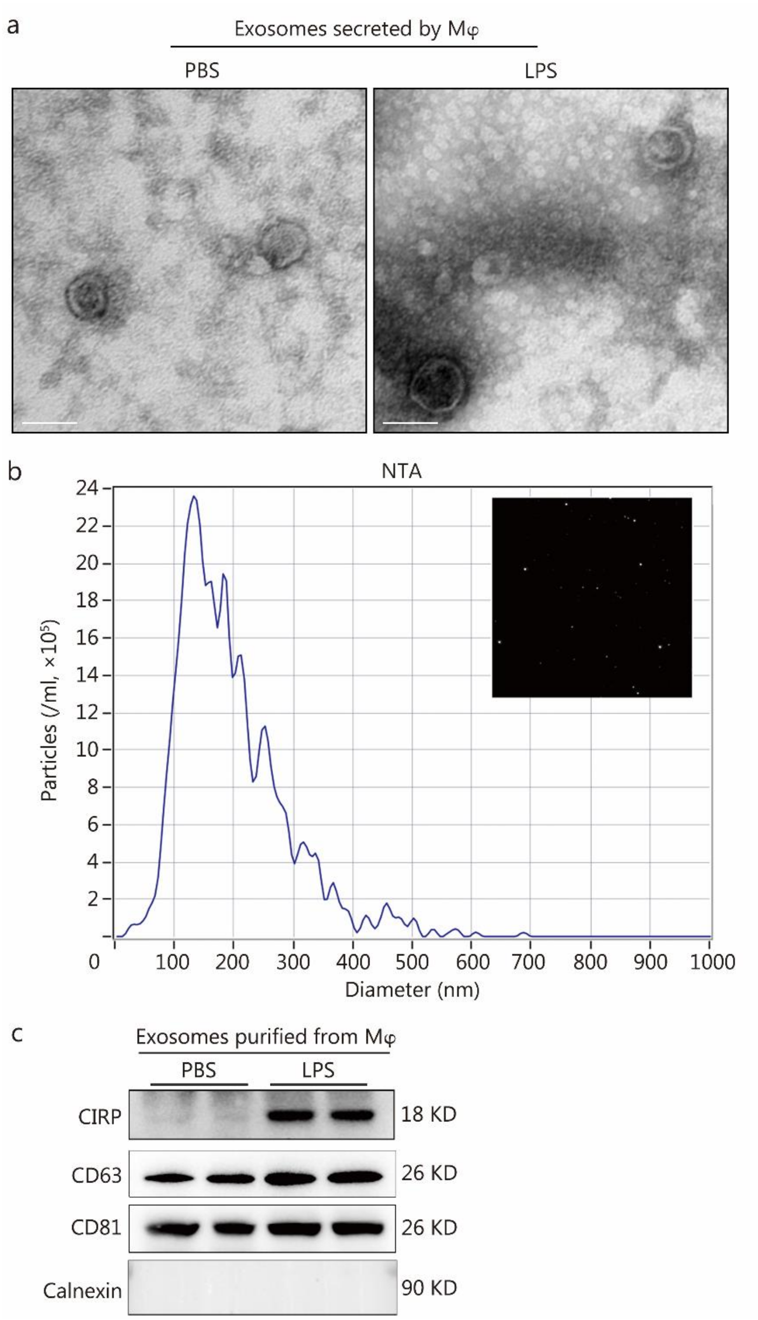

Fig. S3 CIRP is released through the exosome pathway. **a** Transmission electron microscopy images depict the morphology of exosomes isolated from macrophage supernatants (scale bar = 100 nm). **b** Concentration (particles/ml) and modal size (nm) of exosome samples were determined by NTA. **c** Western blotting analysis was used to detect CIRP and exosome markers, including CD63 and CD81, in exosomes purified from macrophages. CIRP cold-inducible RNA-binding protein, NTA nanoparticle tracking analysis, CD63 cluster of differentiation 63, CD81 cluster of differentiation 81, PBS phosphate-buffered saline, LPS lipopolysaccharide

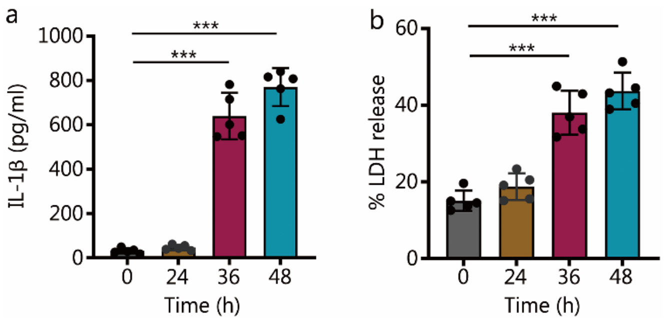

Fig. S4 LPS and rmCIRP induce PANoptosis in MPVECs. **a** Secreted IL-1β measured by ELISA. **b** Cell death quantified by LDH release. The data are presented as the mean ± SD. $^{***}P < 0.001$. MPVECs mouse pulmonary vascular endothelial cells, IL-1β interleukin-1β, ELISA enzyme-linked immunosorbent assay, LDH lactate dehydrogenase, LPS lipopolysaccharide, rmCIRP recombinant mouse cold-inducible RNA-binding protein
