# Supplementary material for: Mechanism of lactic acidemia-promoted pulmonary endothelial cells death in sepsis: role for CIRP-ZBP1-PANoptosis pathway
Source: Mil Med Res. 2024 Oct 28;11:71. doi: 10.1186/s40779-024-00574-z (PMC11514876; doi:10.1186/s40779-024-00574-z)
Supplement: Supplementary file 2 — Additional file 2. [file 40779_2024_574_MOESM2_ESM.pdf]

ZBP1 enriched proteins

Protein	Protein ID	Entry name	Gene	Protein length	Organism	Protein existence
sp|P56873|ZNRD2_MOUSE	P56873	ZNRD2_MOUSE	*Znrd2*	199	Mus musculus	1:Experimental evidence at protein level
sp|Q9QY24|ZBP1_MOUSE	Q9QY24	ZBP1_MOUSE	*Zbp1*	411	Mus musculus	1:Experimental evidence at protein level
sp|P20152|VIME_MOUSE	P20152	VIME_MOUSE	*Vim*	466	Mus musculus	1:Experimental evidence at protein level
sp|Q60932|VDAC1_MOUSE	Q60932	VDAC1_MOUSE	*Vdac1*	296	Mus musculus	1:Experimental evidence at protein level
sp|Q9QZH0|USP7_MOUSE	Q9ESN9	USP7_MOUSE	*USP7*	1102	Mus musculus	1:Experimental evidence at protein level
sp|Q9D6F9|UB2L6_MOUSE	Q9R1X6	UBE2L6_MOUSE	*UBE2L6*	179	Mus musculus	1:Experimental evidence at protein level
sp|P61080|UB2D3_MOUSE	Q9D3K2	UBE2D3_MOUSE	*UBE2D3*	154	Mus musculus	1:Experimental evidence at protein level
sp|P62988|RL40_MOUSE	Q9DCN5	UBA52_MOUSE	*UBA52*	128	Mus musculus	1:Experimental evidence at protein level
sp|P10639|THIO_MOUSE	P10639	THIO_MOUSE	*Txn*	105	Mus musculus	1:Experimental evidence at protein level
sp|P05213|TBA1B_MOUSE	P05213	TBA1B_MOUSE	*Tuba1b*	451	Mus musculus	1:Experimental evidence at protein level
sp|Q8CH72|TRI32_MOUSE	Q8CH72	TRI32_MOUSE	*Trim32*	655	Mus musculus	1:Experimental evidence at protein level
sp|Q6IRU2|TPM4_MOUSE	Q6IRU2	TPM4_MOUSE	*Tpm4*	248	Mus musculus	1:Experimental evidence at protein level
sp|P21107|TPM3_MOUSE	P21107	TPM3_MOUSE	*Tpm3*	285	Mus musculus	1:Experimental evidence at protein level
sp|P58774|TPM2_MOUSE	P58774	TPM2_MOUSE	*Tpm2*	284	Mus musculus	1:Experimental evidence at protein level
sp|P58771|TPM1_MOUSE	P58771	TPM1_MOUSE	*Tpm1*	284	Mus musculus	1:Experimental evidence at protein level
sp|P17751|TPIS_MOUSE	P17751	TPIS_MOUSE	*Tpi1*	249	Mus musculus	1:Experimental evidence at protein level
sp|Q61029|LAP2B_MOUSE	Q61029	LAP2B_MOUSE	*Tmpo*	452	Mus musculus	1:Experimental evidence at protein level
sp|Q9JHJ0|TMOD3_MOUSE	Q9JHJ0	TMOD3_MOUSE	*Tmod3*	352	Mus musculus	1:Experimental evidence at protein level
sp|Q2KN98|CYTSA_MOUSE	Q2KN98	CYTSA_MOUSE	*Specc1l*	1118	Mus musculus	1:Experimental evidence at protein level
sp|P48962|ADT1_MOUSE	P48962	ADT1_MOUSE	*Slc25a4*	298	Mus musculus	1:Experimental evidence at protein level
sp|Q6ZWN5|RS9_MOUSE	Q6ZWN5	RS9_MOUSE	*Rps9*	194	Mus musculus	1:Experimental evidence at protein level
sp|P97461|RS5_MOUSE	P97461	RS5_MOUSE	*Rps5*	204	Mus musculus	1:Experimental evidence at protein level
sp|P62702|RS4X_MOUSE	P62702	RS4X_MOUSE	*Rps4x*	263	Mus musculus	1:Experimental evidence at protein level
sp|P97351|RS3A_MOUSE	P97351	RS3A_MOUSE	*Rps3a*	264	Mus musculus	1:Experimental evidence at protein level
sp|P62980|RS27A_MOUSE	P62269	RS27A_MOUSE	*RPS27A*	84	Mus musculus	1:Experimental evidence at protein level
sp|P62983|RS27A_MOUSE	P62983	RS27A_MOUSE	*Rps27a*	156	Mus musculus	1:Experimental evidence at protein level
sp|P62855|RS26_MOUSE	P62855	RS26_MOUSE	*Rps26*	115	Mus musculus	1:Experimental evidence at protein level
sp|P62852|RS25_MOUSE	P62852	RS25_MOUSE	*Rps25*	125	Mus musculus	1:Experimental evidence at protein level
sp|P62267|RS23_MOUSE	P62267	RS23_MOUSE	*Rps23*	143	Mus musculus	1:Experimental evidence at protein level
sp|P60867|RS20_MOUSE	P60867	RS20_MOUSE	*Rps20*	119	Mus musculus	1:Experimental evidence at protein level
sp|P25444|RS2_MOUSE	P25444	RS2_MOUSE	*Rps2*	293	Mus musculus	1:Experimental evidence at protein level
sp|Q9CZX8|RS19_MOUSE	Q9CZX8	RS19_MOUSE	*Rps19*	145	Mus musculus	1:Experimental evidence at protein level
sp|P62270|RS18_MOUSE	P62270	RS18_MOUSE	*Rps18*	152	Mus musculus	1:Experimental evidence at protein level
sp|P14131|RS16_MOUSE	P14131	RS16_MOUSE	*Rps16*	146	Mus musculus	1:Experimental evidence at protein level

sp|P62264|RS14_MOUSE	P62264	RS14_MOUSE	*Rps14*	151	Mus musculus	1:Experimental evidence at protein level
sp|P62301|RS13_MOUSE	P62301	RS13_MOUSE	*Rps13*	151	Mus musculus	1:Experimental evidence at protein level
sp|P63323|RS12_MOUSE	P63323	RS12_MOUSE	*Rps12*	132	Mus musculus	1:Experimental evidence at protein level
sp|P14869|RLA0_MOUSE	P14869	RLA0_MOUSE	*Rplp0*	317	Mus musculus	1:Experimental evidence at protein level
sp|P62918|RL8_MOUSE	P62918	RL8_MOUSE	*Rpl8*	257	Mus musculus	1:Experimental evidence at protein level
sp|P12970|RL7A_MOUSE	P12970	RL7A_MOUSE	*Rpl7a*	266	Mus musculus	1:Experimental evidence at protein level
sp|P14148|RL7_MOUSE	P14148	RL7_MOUSE	*Rpl7*	270	Mus musculus	1:Experimental evidence at protein level
sp|P47911|RL6_MOUSE	P47911	RL6_MOUSE	*Rpl6*	296	Mus musculus	1:Experimental evidence at protein level
sp|P62892|RL39_MOUSE	P62892	RL39_MOUSE	*Rpl39*	51	Mus musculus	1:Experimental evidence at protein level
sp|P61514|RL37A_MOUSE	P61514	RL37A_MOUSE	*Rpl37a*	92	Mus musculus	1:Experimental evidence at protein level
sp|Q6ZWV7|RL35_MOUSE	Q6ZWV7	RL35_MOUSE	*Rpl35*	123	Mus musculus	1:Experimental evidence at protein level
sp|Q9D1R9|RL34_MOUSE	Q9D1R9	RL34_MOUSE	*Rpl34*	117	Mus musculus	1:Experimental evidence at protein level
sp|P47915|RL29_MOUSE	P47915	RL29_MOUSE	*Rpl29*	160	Mus musculus	1:Experimental evidence at protein level
sp|P14115|RL27A_MOUSE	P14115	RL27A_MOUSE	*Rpl27a*	148	Mus musculus	1:Experimental evidence at protein level
sp|P61358|RL27_MOUSE	P61358	RL27_MOUSE	*Rpl27*	136	Mus musculus	1:Experimental evidence at protein level
sp|P62751|RL23A_MOUSE	P62751	RL23A_MOUSE	*Rpl23a*	156	Mus musculus	1:Experimental evidence at protein level
sp|P62830|RL23_MOUSE	P62830	RL23_MOUSE	*Rpl23*	140	Mus musculus	1:Experimental evidence at protein level
sp|P84099|RL19_MOUSE	P84099	RL19_MOUSE	*Rpl19*	196	Mus musculus	1:Experimental evidence at protein level
sp|P62717|RL18A_MOUSE	P62717	RL18A_MOUSE	*Rpl18a*	176	Mus musculus	1:Experimental evidence at protein level
sp|P35980|RL18_MOUSE	P35980	RL18_MOUSE	*Rpl18*	188	Mus musculus	1:Experimental evidence at protein level
sp|Q9CZM2|RL15_MOUSE	Q9CZM2	RL15_MOUSE	*Rpl15*	204	Mus musculus	1:Experimental evidence at protein level
sp|Q9CR57|RL14_MOUSE	Q9CR57	RL14_MOUSE	*Rpl14*	217	Mus musculus	1:Experimental evidence at protein level
sp|P19253|RL13A_MOUSE	P19253	RL13A_MOUSE	*Rpl13a*	203	Mus musculus	1:Experimental evidence at protein level
sp|P47963|RL13_MOUSE	P47963	RL13_MOUSE	*Rpl13*	211	Mus musculus	1:Experimental evidence at protein level
sp|Q9CXW4|RL11_MOUSE	Q9CXW4	RL11_MOUSE	*Rpl11*	178	Mus musculus	1:Experimental evidence at protein level
sp|P86048|RL10L_MOUSE	P86048	RL10L_MOUSE	*Rpl10l*	214	Mus musculus	1:Experimental evidence at protein level
sp|Q9EP71|RAI14_MOUSE	Q9EP71	RAI14_MOUSE	*Rai14*	979	Mus musculus	1:Experimental evidence at protein level
sp|Q9DBR7|MYPT1_MOUSE	Q9DBR7	MYPT1_MOUSE	*Ppp1r12a*	1029	Mus musculus	1:Experimental evidence at protein level
sp|P62141|PP1B_MOUSE	P62141	PP1B_MOUSE	*Ppp1cb*	327	Mus musculus	1:Experimental evidence at protein level
sp|P52480|KPYM_MOUSE	P52480	KPYM_MOUSE	*Pkm*	531	Mus musculus	1:Experimental evidence at protein level
sp|Q9JM94|PIAS4_MOUSE	Q9QZF2	PIAS4_MOUSE	*PIAS4*	520	Mus musculus	1:Experimental evidence at protein level
sp|Q61753|SERA_MOUSE	Q61753	SERA_MOUSE	*Phgdh*	533	Mus musculus	1:Experimental evidence at protein level
sp|Q5SYD0|MYO1D_MOUSE	Q5SYD0	MYO1D_MOUSE	*Myo1d*	1006	Mus musculus	1:Experimental evidence at protein level
sp|Q60605|MYL6_MOUSE	Q60605	MYL6_MOUSE	*Myl6*	151	Mus musculus	1:Experimental evidence at protein level
sp|Q3THE2|ML12B_MOUSE	Q3THE2	ML12B_MOUSE	*Myl12b*	172	Mus musculus	1:Experimental evidence at protein level
sp|Q8VDD5|MYH9_MOUSE	Q8VDD5	MYH9_MOUSE	*Myh9*	1960	Mus musculus	1:Experimental evidence at protein level
sp|Q6URW6|MYH14_MOUSE	Q6URW6	MYH14_MOUSE	*Myh14*	2000	Mus musculus	1:Experimental evidence at protein level
sp|Q61879|MYH10_MOUSE	Q61879	MYH10_MOUSE	*Myh10*	1976	Mus musculus	1:Experimental evidence at protein level

sp|P11589|MUP2_MOUSE	P11589	MUP2_MOUSE	*Mup2*	180	Mus musculus	1:Experimental evidence at protein level
sp|P97434|MPRIP_MOUSE	P97434	MPRIP_MOUSE	*Mprip*	1024	Mus musculus	1:Experimental evidence at protein level
sp|P48678|LMNA_MOUSE	P48678	LMNA_MOUSE	*Lmna*	665	Mus musculus	1:Experimental evidence at protein level
sp|Q9ERG0|LIMA1_MOUSE	Q9ERG0	LIMA1_MOUSE	*Lima1*	753	Mus musculus	1:Experimental evidence at protein level
sp|Q8VED5|K2C79_MOUSE	Q8VED5	K2C79_MOUSE	*Krt79*	531	Mus musculus	1:Experimental evidence at protein level
sp|Q3UV17|K22O_MOUSE	Q3UV17	K22O_MOUSE	Krt76	594	Mus musculus	1:Experimental evidence at protein level
sp|Q6NXH9|K2C73_MOUSE	Q6NXH9	K2C73_MOUSE	*Krt73*	539	Mus musculus	1:Experimental evidence at protein level
sp|Q9R0H5|K2C71_MOUSE	Q9R0H5	K2C71_MOUSE	*Krt71*	524	Mus musculus	1:Experimental evidence at protein level
sp|P50446|K2C6A_MOUSE	P50446	K2C6A_MOUSE	*Krt6a*	553	Mus musculus	1:Experimental evidence at protein level
sp|Q922U2|K2C5_MOUSE	Q922U2	K2C5_MOUSE	*Krt5*	580	Mus musculus	1:Experimental evidence at protein level
sp|Q6IFX2|K1C42_MOUSE	Q6IFX2	K1C42_MOUSE	*Krt42*	452	Mus musculus	1:Experimental evidence at protein level
sp|Q8VCW2|K1C25_MOUSE	Q8VCW2	K1C25_MOUSE	*Krt25*	446	Mus musculus	1:Experimental evidence at protein level
sp|Q3TTY5|K22E_MOUSE	Q3TTY5	K22E_MOUSE	*Krt2*	707	Mus musculus	1:Experimental evidence at protein level
sp|P19001|K1C19_MOUSE	P19001	K1C19_MOUSE	*Krt19*	403	Mus musculus	1:Experimental evidence at protein level
sp|Q9QWL7|K1C17_MOUSE	Q9QWL7	K1C17_MOUSE	*Krt17*	433	Mus musculus	1:Experimental evidence at protein level
sp|Q9Z2K1|K1C16_MOUSE	Q9Z2K1	K1C16_MOUSE	*Krt16*	469	Mus musculus	1:Experimental evidence at protein level
sp|P02535|K1C10_MOUSE	P02535	K1C10_MOUSE	*Krt10*	570	Mus musculus	1:Experimental evidence at protein level
sp|P04104|K2C1_MOUSE	P04104	K2C1_MOUSE	*Krt1*	637	Mus musculus	1:Experimental evidence at protein level
sp|P01837|IGKC_MOUSE	P01837	IGKC_MOUSE	*Igkc*	107	Mus musculus	1:Experimental evidence at protein level
sp|P01868|IGHG1_MOUSE	P01868	IGHG1_MOUSE	*Ighg1*	324	Mus musculus	1:Experimental evidence at protein level
sp|P01863|GCAA_MOUSE	P01863	GCAA_MOUSE	*Ighg*	330	Mus musculus	1:Experimental evidence at protein level
sp|P63017|HSP7C_MOUSE	P63017	HSP7C_MOUSE	*Hspa8*	646	Mus musculus	1:Experimental evidence at protein level
sp|P08113|ENPL_MOUSE	P08113	ENPL_MOUSE	*Hsp90b1*	802	Mus musculus	1:Experimental evidence at protein level
sp|O35737|HNRH1_MOUSE	O35737	HNRH1_MOUSE	*Hnrnph1*	449	Mus musculus	1:Experimental evidence at protein level
sp|P02088|HBB1_MOUSE	P02088	HBB1_MOUSE	*Hbb-b1*	147	Mus musculus	1:Experimental evidence at protein level
sp|P01942|HBA_MOUSE	P01942	HBA_MOUSE	*Hba*	142	Mus musculus	1:Experimental evidence at protein level
sp|P62806|H4_MOUSE	P62806	H4_MOUSE	*H4c16*	103	Mus musculus	1:Experimental evidence at protein level
sp|P02301|H3C_MOUSE	P02301	H3C_MOUSE	*H3-5*	136	Mus musculus	3:Protein inferred from homology
sp|P10853|H2B1F_MOUSE	P10853	H2B1F_MOUSE	*H2bc15*	126	Mus musculus	1:Experimental evidence at protein level
sp|P0C0S6|H2AZ_MOUSE	P0C0S6	H2AZ_MOUSE	*H2az1*	128	Mus musculus	1:Experimental evidence at protein level
sp|Q8R1M2|H2AJ_MOUSE	Q8R1M2	H2AJ_MOUSE	*H2aj*	129	Mus musculus	1:Experimental evidence at protein level
sp|Q64523|H2A2C_MOUSE	Q64523	H2A2C_MOUSE	H2ac20	129	Mus musculus	1:Experimental evidence at protein level
sp|P43276|H15_MOUSE	P43276	H15_MOUSE	*H1-5*	223	Mus musculus	1:Experimental evidence at protein level
sp|P43274|H14_MOUSE	P43274	H14_MOUSE	*H1-4*	219	Mus musculus	1:Experimental evidence at protein level
sp|P43277|H13_MOUSE	P43277	H13_MOUSE	*H1-3*	221	Mus musculus	1:Experimental evidence at protein level
sp|P15864|H12_MOUSE	P15864	H12_MOUSE	*H1-2*	212	Mus musculus	1:Experimental evidence at protein level
sp|P10922|H10_MOUSE	P10922	H10_MOUSE	*H1-0*	194	Mus musculus	2:Experimental evidence at transcript level
sp|Q9CYL5|GAPR1_MOUSE	Q9CYL5	GAPR1_MOUSE	*Glipr2*	154	Mus musculus	1:Experimental evidence at protein level

sp|P16858|G3P_MOUSE	P16858	G3P_MOUSE	*Gapdh*	333	Mus musculus	1:Experimental evidence at protein level
sp|P56959|FUS_MOUSE	P56959	FUS_MOUSE	*Fus*	518	Mus musculus	1:Experimental evidence at protein level
sp|Q6P9Q4|FHOD1_MOUSE	Q6P9Q4	FHOD1_MOUSE	*Fhod1*	1197	Mus musculus	1:Experimental evidence at protein level
sp|Q8K0E8|FIBB_MOUSE	Q8K0E8	FIBB_MOUSE	*Fgb*	481	Mus musculus	1:Experimental evidence at protein level
sp|P62862|RS30_MOUSE	P62862	RS30_MOUSE	*Fau*	59	Mus musculus	1:Experimental evidence at protein level
sp|Q61545|EWS_MOUSE	Q61545	EWS_MOUSE	*Ewsr1*	655	Mus musculus	1:Experimental evidence at protein level
sp|P17182|ENOA_MOUSE	P17182	ENOA_MOUSE	*Eno1*	434	Mus musculus	1:Experimental evidence at protein level
sp|P58252|EF2_MOUSE	P58252	EF2_MOUSE	*Eef2*	858	Mus musculus	1:Experimental evidence at protein level
sp|P10126|EF1A1_MOUSE	P10126	EF1A1_MOUSE	*Eef1a1*	462	Mus musculus	1:Experimental evidence at protein level
sp|Q61656|DDX5_MOUSE	Q61656	DDX5_MOUSE	*Ddx5*	614	Mus musculus	1:Experimental evidence at protein level
sp|Q9QXS6|DREB_MOUSE	Q9QXS6	DREB_MOUSE	*Dbn1*	706	Mus musculus	1:Experimental evidence at protein level
sp|P16381|DDX3L_MOUSE	P16381	DDX3L_MOUSE	*D1Pas1*	660	Mus musculus	1:Experimental evidence at protein level
sp|Q9JLN9|CUL1_MOUSE	Q9JL24	CUL1_MOUSE	*CUL1*	776	Mus musculus	1:Experimental evidence at protein level
sp|Q9WUM4|COR1C_MOUSE	Q9WUM4	COR1C_MOUSE	*Coro1c*	474	Mus musculus	1:Experimental evidence at protein level
sp|P18760|COF1_MOUSE	P18760	COF1_MOUSE	*Cfl1*	166	Mus musculus	1:Experimental evidence at protein level
sp|P47757|CAPZB_MOUSE	P47757	CAPZB_MOUSE	*Capzb*	277	Mus musculus	1:Experimental evidence at protein level
sp|P47754|CAZA2_MOUSE	P47754	CAZA2_MOUSE	*Capza2*	286	Mus musculus	1:Experimental evidence at protein level
sp|P47753|CAZA1_MOUSE	P47753	CAZA1_MOUSE	*Capza1*	286	Mus musculus	1:Experimental evidence at protein level
sp|Q03265|ATPA_MOUSE	Q03265	ATPA_MOUSE	*Atp5f1a*	553	Mus musculus	1:Experimental evidence at protein level
sp|Q9CPW4|ARPC5_MOUSE	Q9CPW4	ARPC5_MOUSE	*Arpc5*	151	Mus musculus	1:Experimental evidence at protein level
sp|P59999|ARPC4_MOUSE	P59999	ARPC4_MOUSE	*Arpc4*	168	Mus musculus	1:Experimental evidence at protein level
sp|Q9JM76|ARPC3_MOUSE	Q9JM76	ARPC3_MOUSE	*Arpc3*	178	Mus musculus	1:Experimental evidence at protein level
sp|Q9CVB6|ARPC2_MOUSE	Q9CVB6	ARPC2_MOUSE	*Arpc2*	300	Mus musculus	1:Experimental evidence at protein level
sp|Q9WV32|ARC1B_MOUSE	Q9WV32	ARC1B_MOUSE	*Arpc1b*	372	Mus musculus	1:Experimental evidence at protein level
sp|P07356|ANXA2_MOUSE	P07356	ANXA2_MOUSE	*Anxa2*	339	Mus musculus	1:Experimental evidence at protein level
sp|O08583|THOC4_MOUSE	O08583	THOC4_MOUSE	*Alyref*	255	Mus musculus	1:Experimental evidence at protein level
sp|P07724|ALBU_MOUSE	P07724	ALBU_MOUSE	*Alb*	608	Mus musculus	1:Experimental evidence at protein level
sp|Q99JY9|ARP3_MOUSE	Q99JY9	ARP3_MOUSE	*Actr3*	418	Mus musculus	1:Experimental evidence at protein level
sp|P61161|ARP2_MOUSE	P61161	ARP2_MOUSE	*Actr2*	394	Mus musculus	1:Experimental evidence at protein level
sp|P60710|ACTB_MOUSE	P60710	ACTB_MOUSE	*Actb*	375	Mus musculus	1:Experimental evidence at protein level
sp|P62737|ACTA_MOUSE	P62737	ACTA_MOUSE	*Acta2*	377	Mus musculus	1:Experimental evidence at protein level
sp|P01759|HVM15_MOUSE	P01759	HVM15_MOUSE	*Hvm15*	136	Mus musculus	4:Protein predicted
sp|P01638|KV5A6_MOUSE	P01638	KV5A6_MOUSE	*Kv5a6*	115	Mus musculus	4:Protein predicted

Description	Protein probability	Top peptide probability	Combined total peptides	Control intensity	ZBP1 intensity
Protein ZNRD2	0.9869	0.999	1	0	5317528.5
Z-DNA-binding protein 1	1	0.9999	3	0	3.06E+07
Vimentin	1	0.9988	29	0	6.85E+08
Voltage-dependent anion-selective channel prot	0.9756	0.9981	1	0	236860.5
Ubiquitin carboxyl-terminal hydrolase 7	1	0.993	1	0	1.87E+08
Ubiquitin-conjugating enzyme E2 L6	1	0.998	1	0	2.33E+08
Ubiquitin-conjugating enzyme E2 D3	1	0.996	1	0	1.70E+07
60S ribosomal protein L40	1	0.994	1	0	4.83E+09
Thioredoxin	0.8119	0.9825	1	0	4725037
Tubulin alpha-1B chain	1	0.999	4	1.82E+07	3.43E+07
E3 ubiquitin-protein ligase TRIM32	1	0.9999	4	0	2.25E+07
Tropomyosin alpha-4 chain	1	0.9948	11	2.79E+07	5.44E+07
Tropomyosin alpha-3 chain	1	0.9978	12	1.75E+07	2.63E+07
Tropomyosin beta chain	1	0.9975	17	1.78E+07	3.39E+07
Tropomyosin alpha-1 chain	1	0.999	24	0	1.16E+09
Triosephosphate isomerase	0.9797	0.9984	1	0	6170131
Lamina-associated polypeptide 2, isoforms beta/	1	0.9972	4	2.01E+07	8265716.5
Tropomodulin-3	1	0.9959	3	1.16E+07	1.93E+07
Cytospin-A	1	0.9988	12	0	6.39E+07
ADP/ATP translocase 1	1	0.9935	3	0	1.97E+07
40S ribosomal protein S9	1	0.973	5	1.74E+07	2.42E+07
40S ribosomal protein S5	1	0.9904	3	5918982	1.23E+07
40S ribosomal protein S4, X isoform	1	0.9915	4	1.92E+07	2.79E+07
40S ribosomal protein S3a	1	0.999	5	2.65E+07	1.28E+07
40S ribosomal protein S27a	1	0.992	2	0	2.65E+07
Ubiquitin-40S ribosomal protein S27a	1	0.989	3	1.42E+07	1.84E+07
40S ribosomal protein S26	0.9991	0.9811	2	1.09E+07	1.36E+07
40S ribosomal protein S25	1	0.9851	2	1.79E+07	4.91E+07
40S ribosomal protein S23	1	0.9825	3	1.36E+07	1.55E+07
40S ribosomal protein S20	1	0.9962	2	1.81E+07	3.42E+07
40S ribosomal protein S2	1	0.9924	5	2.37E+07	2.62E+07
40S ribosomal protein S19	1	0.9961	5	2.34E+07	5.21E+07
40S ribosomal protein S18	1	0.9984	12	0	2.64E+08
40S ribosomal protein S16	1	0.9973	6	2.24E+07	3.06E+07

40S ribosomal protein S14	1	0.993	2	1.60E+07	2.36E+07
40S ribosomal protein S13	1	0.9958	4	1.22E+07	2.17E+07
40S ribosomal protein S12	0.9872	0.999	1	0	1041558.2
60S acidic ribosomal protein P0	0.9971	0.9683	1	0	3689326.2
60S ribosomal protein L8	1	0.9918	1	0	7546119.5
60S ribosomal protein L7a	1	0.989	2	0	4067124.5
60S ribosomal protein L7	0.9845	0.9988	1	7146621	6070040
60S ribosomal protein L6	1	0.9832	2	0	5654323
60S ribosomal protein L39	0.9999	0.9968	1	2.11E+08	8.20E+08
60S ribosomal protein L37a	0.7158	0.9703	1	4571226	4493192.5
60S ribosomal protein L35	1	0.987	3	2.25E+07	3.60E+07
60S ribosomal protein L34	1	0.9919	3	1.01E+07	1.43E+07
60S ribosomal protein L29	0.9154	0.9929	1	6009767	8779172
60S ribosomal protein L27a	0.9715	0.9977	1	8568689	1.56E+07
60S ribosomal protein L27	0.9997	0.9829	2	7508613	9972414
60S ribosomal protein L23a	1	0.9989	3	1.45E+07	1.55E+07
60S ribosomal protein L23	0.9995	0.9826	2	1.21E+07	1.83E+07
60S ribosomal protein L19	0.9995	0.9909	2	6342246	7401914
60S ribosomal protein L18a	0.7696	0.9775	1	3852416.5	4234556
60S ribosomal protein L18	1	0.9755	3	1.53E+07	5.57E+07
60S ribosomal protein L15	0.998	0.9834	1	5062047	8485027
60S ribosomal protein L14	0.8515	0.9867	1	0	4570797.5
60S ribosomal protein L13a	0.9999	0.9985	2	5421721	8147911
60S ribosomal protein L13	1	0.998	4	1.76E+07	1.97E+07
60S ribosomal protein L11	1	0.9989	3	1.55E+07	2.24E+07
60S ribosomal protein L10-like	0.9989	0.987	1	4268869.5	5361665.5
Ankycorbin	1	0.996	9	3.22E+07	2.91E+07
Protein phosphatase 1 regulatory subunit 12A	1	0.9986	10	0	4.78E+07
Serine/threonine-protein phosphatase PP1-beta	1	0.9985	3	1.12E+07	1.33E+07
Pyruvate kinase PKM	0.9999	0.999	1	1.27E+07	0
E3 SUMO-protein ligase PIAS4	1	0.995	5	0	7.08E+06
D-3-phosphoglycerate dehydrogenase	0.986	0.9989	1	1.27E+07	9059242
Unconventional myosin-Id	1	0.9647	4	1.10E+07	1.85E+07
Myosin light polypeptide 6	1	0.9989	8	4.17E+08	2.67E+09
Myosin regulatory light chain 12B	1	0.9989	6	9.75E+07	3.70E+08
Myosin-9	1	0.999	206	0	5.92E+10
Myosin-14	1	0.9975	20	1.45E+07	4.92E+07
Myosin-10	1	0.999	140	0	1.10E+09

Major urinary protein 2	0.9999	0.9989	9	4.30E+08	0
Myosin phosphatase Rho-interacting protein	1	0.9986	18	0	1.15E+08
Prelamin-A/C	0.9999	0.9939	2	3527627.5	3434901.5
LIM domain and actin-binding protein 1	1	0.9986	8	3.01E+07	3.30E+07
Keratin, type II cytoskeletal 79	0.999	0.9982	6	6.05E+07	2.62E+08
Keratin, type II cytoskeletal 2 oral	0.9967	0.9874	9	3.31E+08	1.97E+09
Keratin, type II cytoskeletal 73	1	0.9984	9	4.22E+08	2.05E+09
Keratin, type II cytoskeletal 71	1	0.9928	10	0	3.88E+07
Keratin, type II cytoskeletal 6A	1	0.9896	13	0	8.32E+07
Keratin, type II cytoskeletal 5	1	0.9976	18	0	4.31E+09
Keratin, type I cytoskeletal 42	1	0.9989	9	5.99E+07	2.34E+08
Keratin, type I cytoskeletal 25	0.9992	0.9761	2	0	1.74E+07
Keratin, type II cytoskeletal 2 epidermal	1	0.9966	11	0	1.65E+09
Keratin, type I cytoskeletal 19	0.9989	0.9756	5	0	2756221.5
Keratin, type I cytoskeletal 17	0.9996	0.9988	7	1.31E+08	1.03E+09
Keratin, type I cytoskeletal 16	1	0.9987	7	0	4.67E+07
Keratin, type I cytoskeletal 10	1	0.9987	7	1.11E+09	6.46E+09
Keratin, type II cytoskeletal 1	1	0.9984	8	5.11E+08	2.79E+09
Immunoglobulin kappa constant	1	0.9989	6	1.77E+08	7.87E+09
Ig gamma-1 chain C region secreted form	1	0.995	8	0	2.10E+09
Ig gamma-2A chain C region, A allele	1	0.999	7	2.74E+08	0
Heat shock cognate 71 kDa protein	0.987	0.999	1	4193478	0
Endoplasmin	0.9854	0.9989	1	0	3629741.8
Heterogeneous nuclear ribonucleoprotein H	0.983	0.9987	1	5674244.5	6091993
Hemoglobin subunit beta-1	1	0.994	3	1.36E+07	7.46E+07
Hemoglobin subunit alpha	1	0.9989	4	3.81E+07	1.01E+08
Histone H4	1	0.9984	6	5.37E+08	3.57E+08
Histone H3.3C	1	0.9731	5	0	1.34E+08
Histone H2B type 1-F/J/L	1	0.9983	5	3.66E+08	2.81E+08
Histone H2A.Z	0.9165	0.9842	3	0	2774139.8
Histone H2A.J	1	0.999	4	2.58E+08	2.43E+08
Histone H2A type 2-C	0.9933	0.9748	4	4090684.2	0
Histone H1.5	1	0.9828	3	7465457	1.07E+07
Histone H1.4	1	0.9971	7	3.45E+07	3.69E+07
Histone H1.3	0.9999	0.9975	8	1.86E+07	6.69E+07
Histone H1.2	0.9997	0.9962	9	2.54E+07	2.75E+07
Histone H1.0	1	0.9833	2	8960925	7918984
Golgi-associated plant pathogenesis-related pro	0.9748	0.998	1	3186382.2	3353871.5

Glyceraldehyde-3-phosphate dehydrogenase	1	0.9986	5	3.47E+07	5.22E+07
RNA-binding protein FUS	0.9223	0.9935	1	0	3303288.5
FH1/FH2 domain-containing protein 1	1	0.9978	11	0	1.50E+08
Fibrinogen beta chain	1	0.9938	2	1.67E+07	0
40S ribosomal protein S30	0.9999	0.9866	2	3.03E+07	7.07E+07
RNA-binding protein EWS	0.937	0.9948	1	5002479	3254561.5
Alpha-enolase	1	0.999	2	1.67E+07	4526471.5
Elongation factor 2	0.9871	0.999	1	1.21E+07	1.59E+07
Elongation factor 1-alpha 1	1	0.999	10	1.01E+08	2.03E+08
Probable ATP-dependent RNA helicase DDX5	1	0.9957	2	5996839	4519554
Drebrin	0.8287	0.9843	1	2880247	0
Putative ATP-dependent RNA helicase Pl10	1	0.991	2	0	1.30E+07
Cullin-1	1	0.997	7	0	5.73E+06
Coronin-1C	1	0.9972	3	6983904	6555203
Cofilin-1	0.9663	0.9973	1	3871088.8	5991808
F-actin-capping protein subunit beta	1	0.9959	2	2.33E+07	3.46E+07
F-actin-capping protein subunit alpha-2	1	0.9988	5	4.25E+07	6.18E+07
F-actin-capping protein subunit alpha-1	1	0.9979	5	9633838	8339953
ATP synthase subunit alpha, mitochondrial	1	0.9976	1	6347893	1.31E+07
Actin-related protein 2/3 complex subunit 5	1	0.9648	2	5230575.5	7316937.5
Actin-related protein 2/3 complex subunit 4	1	0.9934	6	4.76E+07	8.08E+07
Actin-related protein 2/3 complex subunit 3	1	0.9935	1	4796796.5	4646833
Actin-related protein 2/3 complex subunit 2	1	0.993	5	0	2.57E+07
Actin-related protein 2/3 complex subunit 1B	1	0.9987	4	1.66E+07	2.36E+07
Annexin A2	0.9224	0.9936	1	3524733	4340281
THO complex subunit 4	0.9601	0.9968	1	3115706.2	3183528.2
Albumin	1	0.9984	3	1.09E+08	1.77E+08
Actin-related protein 3	0.9992	0.9817	2	1.12E+07	1.45E+07
Actin-related protein 2	1	0.9983	1	4545862	4552789.5
Actin, cytoplasmic 1	1	0.999	22	0	4.05E+10
Actin, aortic smooth muscle	1	0.9986	16	4.91E+07	5.17E+07
Ig heavy chain V region BCL1	0.9996	0.9791	1	0	3.33E+07
Ig kappa chain V-V region L6 (Fragment)	1	0.999	1	4.70E+07	0
